
JOURNAL INFORMATION
==============================
NLM Title Abbreviation: Insights Imaging
Journal ID: Insights Imaging
NLM Journal ID: 101532453

Insights into Imaging

EISSN: 1869-4101
Publisher: Springer

ARTICLE INFORMATION
==============================
PMCID: PMC8200314
PMID: 34121138
DOI: 10.1186/s13244-021-01015-4
Article ID: 1015
Article version: 2
Subjects: Meeting Abstracts

ESGAR 2021 Book of Abstracts

Electronic publication date: 2021 Jun 13
Publication date: 2021 Jun
Volume: 12
Issue: Suppl 3
Issue sponsor: Publication costs were not supported by sponsorship.
Electronic Location ID: 74
Copyright: © The Author(s) 2021
Copyright year: 2021
License: Open Access This article is licensed under a Creative Commons Attribution 4.0 International License, which permits use, sharing, adaptation, distribution and reproduction in any medium or format, as long as you give appropriate credit to the original author(s) and the source, provide a link to the Creative Commons licence, and indicate if changes were made. The images or other third party material in this article are included in the article’s Creative Commons licence, unless indicated otherwise in a credit line to the material. If material is not included in the article’s Creative Commons licence and your intended use is not permitted by statutory regulation or exceeds the permitted use, you will need to obtain permission directly from the copyright holder. To view a copy of this licence, visit http://creativecommons.org/licenses/by/4.0/.
License URL: https://creativecommons.org/licenses/by/4.0/

Conference name: ESGSR Virtual Meeting 2021
Conference location: Virtual
Conference date: 15-18 June 2021
issue-copyright-statement: © European Society of Radiology (ESR) 2021

==============================

European Society
ESGAR
Gastrointestinal and Abdominal Radiology

### Insights into Imaging

Education and strategies in European radiology

ESGAR 2021 Book of Abstracts / Volume 12 / Supplement 3 / June 2021

ESGAR 2021 / June 15 – 18 / VIRTUAL CONGRESS
32nd Annual Meeting and Postgraduate Course

ESR
European Society of Radiology

European Society
ESGAR
Gastrointestinal and Abdominal Radiology

### ESGAR VIRTUAL MEETING 2021

June 15-18

let's stay...
**#connected**

*ORGANISING SECRETARIAT*

**Central ESGAR Office**
Esslinggasse 2/3
AT – 1010 Vienna
Phone: +43 1 535 89 27
E-Mail: office@esgar.org

*WEBSITE*

www.esgar.org

### CME CREDITS

The "ESGAR European Society of Gastrointestinal and Abdominal Radiology" (or) "ESGAR 2021 – 32nd ANNUAL MEETING AND POSTGRADUATE COURSE" is accredited by the European Accreditation Council for Continuing Medical Education (EACCME) to provide the following CME activity for medical specialists. The EACCME is an institution of the European Union of Medical Specialists (UEMS), www.uems.net.

The ESGAR 32nd Annual Meeting and Postgraduate Course, the 2nd Virtual Meeting, 15/06/2021-18/06/2021 has been accredited by the European Accreditation Council for Continuing Medical Education (EACCME®) with 27 European CME credits (ECMEC®s). Each medical specialist should claim only those hours of credit that he/she actually spent in the educational activity.

### CONTRIBUTING SOCIETIES

ASAR – Asian Society of Abdominal Radiology, Since 2007

SAR – Society of Abdominal Radiology

### SPONSORS

ESGAR wishes to gratefully acknowledge the support of its Corporate Members:

BAYER    BRACCO – LIFE FROM INSIDE    GE Healthcare    Guerbet – Contrast for Life

The Final Programme of ESGAR 2021 is available on the ESGAR Website www.esgar.org

Date of publishing: June 2021

Insights Imaging (2021) 12 (Suppl 3): 74
https://doi.org/10.1186/s13244-021-01015-4
Published Online: 14 June 2021

### TABLE OF CONTENTS

| | |
|---|---|
| Scientific Sessions, Wednesday, June 16 (SSL 1.1 – SSL 1.6) | 8-9 |
| Scientific Sessions, Thursday, June 17 (SSL 2.1 – SSL 2.7) | 10-11 |
| Scientific Sessions, Friday, June 18 (SSL 3.1 – SSL 3.7) | 12-13 |
| Scientific Sessions, On Demand (SSD 1.1 – SSD 6.10) | 14-29 |
| Authors' Index | 31-33 |

European Society
ESGAR
Gastrointestinal and Abdominal Radiology

### ESGAR EXECUTIVE COMMITTEE

PRESIDENT
*R.G.H. Beets-Tan, Amsterdam/NL*

PRESIDENT-ELECT
*A. Laghi, Rome/IT*

VICE PRESIDENT
*M. Zins, Paris/FR*

SECRETARY/TREASURER
*S.A. Taylor, London/UK*

PROGRAMME COMMITTEE CHAIR
*M. Zins, Paris/FR*

EDUCATION COMMITTEE CHAIR
*S. Jackson, Plymouth/UK*

MEMBERSHIP COMMITTEE CHAIR
*G. Brancatelli, Palermo/IT*

RESEARCH COMMITTEE CHAIR
*J. Stoker, Amsterdam/NL*

WORKSHOP COMMITTEE CHAIR
*M.A. Bali, Brussels/BE*

PRE-MEETING PRESIDENT
*L. Curvo-Semedo, Coimbra/PT*

PRE-PRE-MEETING PRESIDENT
*L. Blomqvist, Stockholm/SE*

MEMBERS AT LARGE
*A. Ba-Ssalamah, Vienna/AT*
*S. Gourtsoyianni, Athens/GR*
*V. Vilgrain, Clichy/FR*
*C.J. Zech, Basel/CH*

ESGAR EXECUTIVE DIRECTOR
*B. Lindlbauer, Vienna/AT*

### ESGAR 2021 MEETING PRESIDENT

*Dr. Marc Zins*
*Hôpital Saint-Joseph*
*Department of Radiology*
*185 Rue Raymond Losserand*
*Paris, France*

### ESGAR 2021 PROGRAMME COMMITTEE

CHAIRMAN
*M. Zins, Paris/FR*

MEMBERS
*M.A. Bali, Brussels/BE*
*A. Ba-Ssalamah, Vienna/AT*
*R.G.H. Beets-Tan, Amsterdam/NL*
*L. Blomqvist, Stockholm/SE*
*G. Brancatelli, Palermo/IT*
*L. Curvo-Semedo, Coimbra/PT*
*S. Gourtsoyianni, Athens/GR*
*S. Jackson, Plymouth/UK*
*A. Laghi, Rome/IT*
*J. Stoker, Amsterdam/NL*
*S.A Taylor, London/UK*
*V. Vilgrain, Clichy/FR*
*C.J. Zech, Basel/CH*

*O. Akhan, Ankara/TR*
*C. Aubé, Angers/FR*
*M.A. Bali, Brussels/BE*
*I. Bargellini, Pisa/IT*
*T.V. Bartolotta, Palermo/IT*
*A. Ba-Ssalamah, Vienna/AT*
*N. Bastati-Huber, Vienna/AT*
*R.G.H. Beets-Tan, Amsterdam/NL*
*E. Biscaldi, Genoa/IT*
*I. Boulay Coletta, Paris/FR*
*G. Brancatelli, Palermo/IT*
*R. Cannella, Palermo/IT*
*K. Cortis, Msida/MT*
*N. Courcoutsakis, Alexandroupolis/GR*
*L. Crocetti, Pisa/IT*
*L. Curvo-Semedo, Coimbra/PT*
*G. D'Assignies, La Roche-sur-Yon/FR*
*M. Dioguardi Burgio, Clichy/FR*
*A. Dohan, Paris/FR*
*M. D'Onofrio, Verona/IT*
*H. Fenlon, Dublin/IE*
*A. Furlan, Pittsburgh, PA/US*
*Y. Gandon, Rennes/FR*
*V. Goh, London/UK*
*M. Gollub, New York, NY/US*
*S. Gourtsoyianni, Athens/GR*
*S. Gryspeerdt, Roeselare/BE*
*L. Guimarães, Porto/PT*
*J.A. Guthrie, Leeds/UK*
*S. Halligan, London/UK*
*T. Helmberger, Munich/DE*
*F. Iafrate, Rome/IT*
*D. Ippolito, Monza/IT*
*S. Jackson, Plymouth/UK*
*M. Karcaaltincaba, Ankara/TR*
*A. Laghi, Rome/IT*
*D. Lambregts, Amsterdam/NL*
*M. Laniado, Dresden/DE*
*P. Lebert, Lille/FR*
*J.M. Lee, Seoul/KR*
*M. Lewin, Villejuif/FR*
*O. Lucidarme, Paris/FR*
*M. Maas, Amsterdam/NL*
*A. Madureira, Porto/PT*
*D. Malone, Dublin/IE*
*T. Mang, Vienna/AT*
*V. Maniatis, Sønderborg/DK*
*D. Marin, Durham, NC/US*
*L. Martí-Bonmatí, Valencia/ES*
*C. Matos, Lisbon/PT*
*Y. Menu, Paris/FR*
*G. Morana, Treviso/IT*
*G.H. Mostbeck, Vienna/AT*
*S. Mulé, Créteil/FR*
*E. Neri, Pisa/IT*
*A. Palkó, Szeged/HU*
*P. Paolantonio, Rome/IT*
*N. Papanikolaou, Lisbon/PT*
*S. Pötter-Lang, Vienna/AT*
*R. Pozzi Mucelli, Verona/IT*
*P. Prassopoulos, Thessaloniki/GR*
*E. Quaia, Padua/IT*
*G.A. Rollandi, Genoa/IT*
*M. Ronot, Clichy/FR*
*W. Schima, Vienna/AT*
*A. Schreyer, Regensburg/DE*
*O. Seror, Bondy/FR*
*S. Skehan, Dublin/IE*
*M. Staunton, Cork/IE*
*S. Stojanovic, Novi Sad/RS*
*J. Stoker, Amsterdam/NL*
*A. Taibbi, Palermo/IT*
*B. Taouli, New York, NY/US*
*J.P. Tasu, Poitiers/FR*
*S.A. Taylor, London/UK*
*S. Terraz, Geneva/CH*
*D. Tolan, Leeds/UK*
*C. Triantopoulou, Athens/GR*
*V. Valek, Brno-Bohunice/CZ*
*F. Vernuccio, Palermo/IT*
*V. Vilgrain, Clichy/FR*
*M.-P. Vullierme, Clichy/FR*
*M. Wagner, Paris/FR*
*G. Zamboni, Verona/IT*
*C.J. Zech, Basel/CH*
*M. Zins, Paris/FR*

European Society
ESGAR
Gastrointestinal and Abdominal Radiology

**13:15 - 14:15** **CHANNEL 3**

*Scientific Session Live SSL 1*

### Best papers in Artificial intelligence and radiomics

### SSL 1.1

### Automated deep learning CT-based spleen volume segmentation: defining normal and splenomegaly for clinical practice

A. Perez[1], V. Noe-Kim[1], M. Lubner[1], R. Summers[2], P.J. Pickhardt[1]; [1]Madison, WI/US, [2]Bethesda, MD/US

**Purpose:** Imaging assessment for splenomegaly is not well defined and currently utilizes suboptimal unidimensional measures. Spleen volume provides a more direct measure for organ enlargement. We applied a validated deep learning artificial intelligence tool that automatically segments the spleen for organ volume and sought to establish thresholds for splenomegaly.
**Material and methods:** Spleen volumes were successfully derived with the deep learning tool from all 8853 asymptomatic outpatient adults (mean age 56.3 years; 4223M/4630F) who underwent MDCT for either colorectal screening (n=7688 unenhanced) or renal donor evaluation (n=1165, contrast-enhanced). Linear regression analysis was utilized to assess major patient-specific determinate(s) of spleen volume amongst age/sex/height/weight/body surface area. Threshold for splenomegaly was set at two standard deviations above the mean for the final modeled equation. Accuracy of automated linear measures was assessed. Unenhanced spleen volumes were standardized to a post-contrast equivalent, reflecting a small but constant 7.6% correction.
**Results:** Mean standardized automated spleen volume was 216±100 ml and demonstrated a normal distribution. Patient weight was the major determinant of spleen volume. From this, a linear weight-based splenomegaly threshold volume (in ml) = 3.0x(Wt)+127. Above 125 kg, the splenomegaly threshold was kept at 128 ml. Linear measures demonstrated only moderate performance for identifying volume-defined splenomegaly. For example, a craniocaudal threshold of 10 cm was 95% sensitive and 75% specific.
**Conclusion:** We derived a simple, weight-based threshold for splenomegaly using an automated spleen volume tool. If further validated in larger healthy and diseased cohorts, this approach could provide a more objective and accurate measure of spleen size from CT.

### SSL 1.2

### Automatized hepatic tumor volume analysis of neuroendocrine liver metastases by gadoxetic acid MRI: a deep learning model to support multidisciplinary cancer conference decision-making

U. Fehrenbach[1], S. Xin[1], A. Hartenstein[1], T.A. Auer[1], F. Dräger[1], K. Froböse[1], H. Jann[1], M. Mogl[1], H. Amthauer[1], D. Geisel[1], T. Denecke[2], B. Wiedenmann[1], T. Penzkofer[1]; [1]Berlin/DE, [2]Leipzig/DE

**Purpose:** Rapid quantification of liver metastasis for diagnosis and follow-up is an unmet medical need in patients with secondary liver malignancies. We present a 3D-quantification model of neuroendocrine liver metastases (NELM) using gadoxetic acid (Gd-EOB)-enhanced MRI as a useful tool for multidisciplinary cancer conferences (MCCs).
**Material and methods:** 149 patients with neuroendocrine neoplasms (NEN) fulfilled our inclusion criteria, from which 278 Gd-EOB MRI scans were acquired. Manual segmentation of NELM was performed in hepatobiliary phase sequences. Segmentations and scans were used to train a neural network with U-Net architecture to segment both liver and liver metastases. The clinical usefulness was evaluated in another 33 patients who were discussed in our MCC and received a Gd-EOB MRI both at baseline as well as follow-up examination (n=66) over a time period of 12 months. Model measurements (NELM volume and hepatic tumor load (HTL)) and the corresponding absolute ($\Delta_{abs}$NELM; $\Delta_{abs}$HTL) and relative changes ($\Delta_{rel}$NELM; $\Delta_{rel}$HTL) between baseline and follow-up were compared to MCC decision (therapy success/failure).
**Results:** The model's segmentations showed high overlap for NELM and livers (dice coefficient: 0.75/0.95, respectively) with higher coefficients in larger NELM volume (0.80 vs. 0.71; p=0.002). MCC decisions were significantly differentiated by all response variables ($\Delta_{abs}$NELM; $\Delta_{abs}$HTL; $\Delta_{rel}$NELM; $\Delta_{rel}$HTL) (p<0.001). $\Delta_{rel}$NELM and $\Delta_{rel}$HTL showed optimal discrimination between therapy success or failure (AUC: 1.000; p<0.001).
**Conclusion:** The segmentation model shows high accuracy in 3D volumetry of NELM and determination of HTL by Gd-EOB MRI. The model's measurements correlated well with the MCC's evaluation of therapeutic response.

### SSL 1.3

### Correlation of histologic, imaging, and artificial intelligence features in non-alcoholic fatty liver disease patients, derived from gadoxetic acid-enhanced MRI: a proof-of-concept study

N. Bastati-Huber[1], M. Perkonigg[1], S. Pötter-Lang[1], A. Beer[1], A. Herold[1], I. Shchekoturov[2], J.C. Hodge[1], G. Langs[1], A. Ba-Ssalamah[1]; [1]Vienna/AT, [2]Moscow/RU

**Purpose:** To compare the ability of unsupervised deep clustering (UDC), fat fraction (FF) and relative liver enhancement (RLE), derived from gadoxetic acid (Gd-EOB-DTPA)-enhanced MRI (Gd-EOB-DTPA-MRI), to distinguish simple steatosis from non-alcoholic steatohepatitis (NASH), using histology as the gold standard.
**Material and methods:** Prospectively, 46 non-alcoholic fatty liver disease (NAFLD) patients underwent 3T MRI. Histology assessed steatosis, inflammation, ballooning, and fibrosis. UDC was trained to extract 10 distinct texture clusters per sequence on unenhanced and Gd-EOB-DTPA-enhanced T1-weighted hepatobiliary phase (T1-Gd-EOB-DTPA-HBP) and then on in- and opposed-phased images. Also, RLE and FF were quantified on identical sequences. Differences of these parameters between NASH and simple steatosis were evaluated using $\chi^2$ test and t test, respectively. Univariate and multivariate analyses were performed using linear regression and random forest classifier to identify association(s) between various histological NAFLD features and RLE, FF, plus UDC patterns and then determine predictors able to distinguish simple steatosis from NASH. Receiver operating characteristic curves assessed diagnostic performance of UDC, RLE, and FF.
**Results:** UDC-derived features from in and opposed phases and unenhanced and T1-Gd-EOB-DTPA-HBP distinguished NASH from simple steatosis (p=<0.001 and p=0.02, respectively), with an accuracy of 85% and 80%, respectively. Likewise, RLE and FF distinguished NASH from simple steatosis (p=<0.001 and p=0.004, respectively), with an accuracy of 83% and 78%, respectively. On multivariate regression analysis, RLE and FF correlated only with fibrosis (p=0.040) and steatosis (p=<0.001), respectively. Conversely, UDC features, using random forest classifier predictors, correlated with all histologic NAFLD components.
**Conclusion:** Our preliminary results showed that UDC, RLE and FF could independently separate NASH and simple steatosis. UDC may predict all histologic NAFLD components.

### SSL 1.4

### Adhesive-related small bowel obstruction: deep learning for the automatic detection of the transition zone on CT scans

Q. Vanderbecq[1], R. Ardon[1], A. De Reviers[1], C. Ruppli[1], A. Dallongeville[1], I. Boulay Coletta[1], G. D'Assignies[2], M. Zins[1]; [1]Paris/FR, [2]La Roche-sur-Yon/FR

**Purpose:** To train machine learning models to locate the transition zone of adhesive-related small bowel obstruction (SBO) in CT scans.
**Material and methods:** We used 562 CT examinations performed between January 2005 and July 2018 from 404 patients with a diagnosis of adhesive-related SBO. Annotation of the transition zone was performed by experimented radiologists and trained residents with bounding boxes. A pretrained model was used to extract abdominal and pelvic areas as preprocessing. We modeled the localization problem of the transition zone as a binary classification problem by splitting the abdominal and pelvic areas into 125 patches and trained a neural network model to classify each patch as containing or not a transition zone. We coupled this with a trained probabilistic estimation of the presence of a transition zone in each patch. The models were evaluated first using area under the curve metric (AUC). Second, to evaluate the clinical benefit, we measured the ratio of abdomen volume classified as containing the transition zone for a given localization success rate.
**Results:** SBO transition zone had a higher probability to locate in the hypogastric region. The coupled classification network and spatial probability estimation obtained an AUC of 0.94. For a 15% volume ratio classified as the transition zone, the probability to contain the transition zone is 92%.
**Conclusion:** Modeling the SBO localization problem by coupling CNN classification and probabilistic localization estimation shows the way to a possible automatic detection of the transition zone, a complex radiological task with a high clinical impact.

### SSL 1.5

### Overall survival of colorectal cancer patients stratified by metastatic disease patterns identified on CT report using natural language processing

P.I. Causa Andrieu[1], K. Lupton[2], V. Sevilimedu[1], N. Huy[1], M. Maya[1], K. Batch[2], F. Zulkernine[2], G. Lior[1], N. Gangai[1], A. Simpson[2], J. Golia Pernicka[1], R. Do[1]; [1]New York, NY/US, [2]Kingston, ON/CA

**Purpose:** To compare the overall survival (OS) of colorectal cancer patients (CRC) with metastases reported on chest, abdominal, and pelvic CT scans (CT-CAP), staged by the involved number of sites according to the American Joint Committee Cancer-8th Edition.
**Material and methods:** CT-CAP reports from 2009 to 2019 were extracted. 2219 reports manually annotated by radiologists were used to train a natural language processing (NLP) model to predict the presence or absence of metastases in lungs, pleura, thoracic nodes, liver, spleen, adrenal glands, abdominopelvic nodes, pelvic organs, peritoneum, and bones. NLP accuracy was evaluated on a validation set of 448 reports. Survival curves were generated by Kaplan–Meier analysis; OS was compared between CRC without (M0) or with metastatic disease to a single site (M1a), two or more sites (M1b), or to the peritoneum (M1c).
**Results:** 10,495 CRC were included, 46.52% women, mean [SD] age of 60.16 [13.68] years. NLP accuracy ranged from 90% to 99% for every organ. Metastases were most frequent in liver and lungs (>20%), followed by peritoneum and nodes (9%–17%) and rare in pleura, pelvic organs, and spleen (0.4%–4.0%). Compared to 4,266 M0, each M1 substage was significantly associated with increased mortality risk (p<0.001): M1a (n=3,047), HR 4.75, 95% CI 4.27–5.28; M1b (n=1,802), HR 12.0, 95% CI 10.8–13.3; and M1c (n=1,380), HR 13.2, 95% CI 11.8–14.8.
**Conclusion:** Increased number of metastatic sites and peritoneal metastases identified by NLP in CT-CAP reports are associated with increased CRC mortality. NLP has the potential of extracting large-scale metastatic phenotypes from radiology reports in cancer patients.

### SSL 1.6

### Artificial intelligence-based detection of pneumoperitoneum on abdominal CT scans in patients presenting with acute abdominal pain: a retrospective clinical cohort diagnostic test accuracy study

M.W. Brejnebøl[1], Y.J.W. Nielsen[2], O. Taubmann[3], E. Eibenberger[3], F.C. Müller[2]; [1]Copenhagen/DK, [2]Herlev/DK, [3]Forchheim/DE

**Purpose:** We investigated a novel artificial intelligence (AI) algorithm for the detection of pneumoperitoneum in a clinical patient population.
**Material and methods:** This retrospective diagnostic test accuracy study used a consecutive patient cohort from the acute high-risk abdominal patient population at Herlev and Gentofte Hospital, Denmark, between January 1, 2019, and April 30, 2020. As reference standard, all cases were rated for pneumoperitoneum (subgroups: none, small, medium, and large amounts). Two radiology residents initially read all cases with a radiology consultant reviewing scans with pneumoperitoneum and 50 randomly selected cases without. The index test was a novel AI algorithm based on a sliding window approach with deep recurrent neural network at its core. The primary outcome was the area under the curve (AUC) of the receiver operating characteristic.
**Results:** Of 330 included patients (median age 68 years (range 19–100; 179 women)), 30 patients (9%) had pneumoperitoneum (large: 16, moderate: 7, small: 7). In the entire group, the AUC was 0.78 (95% CI 0.66–0.88). At 99% specificity, sensitivity was 53% (16/30) and positive likelihood ratio was 53 (95% CI 16–173). When excluding small amounts (diameter <5 mm), the AUC increased to 0.85 (95% CI 0.73–0.94). At 99% specificity, sensitivity was 65% (15/23) and positive likelihood ratio was 65 (95% CI 20–209).
**Conclusion:** A novel AI algorithm identified pneumoperitoneum on CT scans in a clinical setting with moderate sensitivity and very low false-positive rate, supporting its role for ruling in pneumoperitoneum.

13:15 - 14:15 CHANNEL 3

*Scientific Session Live SSL 2*

### Best papers in GI Cancer Imaging

### *SSL 2.1*

**Prognostic significance of MRI-identified extramural vascular invasion, tumour deposits and mesorectal nodes in locally advanced rectal cancer**

A. Chandramohan, R. D'Souza, H. Yezzaji, A. Eapen, B. Simon, R. John, D. Masih, A. Singh, M.R. Jesudason, T.S. Ram, R. Mittal; Vellore/IN

**Purpose:** To study the prognostic significance of MRI-identified extramural vascular invasion (EMVI), tumour deposits (TD), significant mesorectal nodes (LN) and pelvic sidewall disease (PSW) in locally advanced rectal cancer.
**Material and methods:** This is an institutional review board-approved study of patients with non-metastatic locally advanced rectal adenocarcinoma treated with neo-adjuvant long-course chemoradiotherapy and surgery. Staging and restaging MRI was reviewed for EMVI, TD, LN and PSW by a radiologist blinded to the patient's outcome. Interobserver agreement (IOA) between four radiologists was studied in an anonymised subset. Outcome data were obtained from a prospectively maintained database. Prognostic significance of imaging findings was assessed using Kaplan–Meier analysis and Cox proportional hazard model.
**Results:** 297 (186 males) patients with mean age of 47.3 (SD14.4) years were included. Majority had T3 (n=206) or T4 (n=59) stage disease. Mean duration of follow-up was 49.3+/-25 months (6.6–101 months). Staging MRI had EMVI, TD, LN and PSW in 49.5%, 47.5%, 47.4% and 11.4%, respectively, and re-staging MRI had these in 31.3%, 31.6%, 15.8% and 6.1%, respectively. Disease-free survival (DFS) was adversely affected by TD on staging MRI (HR=2.44(1.47–3.75); EMVI (HR=2.32(1.44–3.75) and TD (HR=2.53(1.57–4.09) on restaging MRI, p=0.001. Similarly, overall survival was adversely affected by TD (HR=3.01(1.53–5.92) and PSW (HR=3.26(1.59–6.69) on staging MRI and EMVI (HR=2.74(1.47–5.11) and TD (HR=2.66(1.43–4.95) on restaging MRI, p=0.001. IOA was fair with multirater Kappa of 0.436, 0.439 and 0.321 for EMVI, TD and LN, respectively, p<0.001.
**Conclusion:** EMVI and TD on staging and restaging MRI adversely affect disease-free and overall survival of rectal cancer patients with fair IOA.

### *SSL 2.2*

**Applying the TNM (8th ed) classification for the radiological staging of rectal cancer: preliminary results from a global online survey**

N. Bogveradze[1], R.G.H. Beets-Tan[1], G.L. Beets[1], M.J. Lahaye[1], M. Maas[1], N.E. Khababi[1], S. Kakhadze[2], B. Jeremic[2], D. Lambregts[1]; [1]Amsterdam/NL, [2]Tbilisi/GE

**Purpose:** To identify any potential problem areas in the applicability of the TNM8 classification for the radiological staging of rectal cancer.
**Material and methods:** A web-based survey including 52 cases/questions related to various staging TNM8 staging scenarios for (colo)rectal cancer was circulated among radiologists/radiology residents and clinical colleagues worldwide. Each case was based on a single MRI (or CT) image + schematic representation and a detailed description of the imaging findings. Responses were analyzed using descriptive statistics and correlated with nature of practice (academic/non-academic/cancer-referral center), profession (radiologists/non-radiologists), and experience level. Items with <80% consensus were classified as potential problem areas.
**Results:** To date, the survey has been completed by 311 respondents (from 31 countries), of which 82% are radiologists/radiology residents with an experience level in rectal imaging of >10 years (32%), 5–10 years (32%) and <5 years (36%), respectively. Respondents originated from academic/non-academic/cancer-referral centers in 48%/29%/23%, respectively. Main problem areas (<80% consensus) included the following: 1. Effect of sphincter invasion on T-stage (39–68% consensus). 2. Mesorectal fascia versus peritoneal invasion in tumours above the peritoneal reflection (52% consensus). 3.Differentiation between lymph nodes and tumour deposits (43% consensus). 4.Differentiation of regional (N) versus non-regional (M) nodes (57–68% consensus). Items 1 and 3 showed significant correlations with experience level and/or profession (p=0.01).
**Conclusion:** With this survey, we have identified several potential problem areas that can serve as a basis for discussion to produce more practical guidelines on the radiological application of the TNM8 specified for rectal cancer ultimately aiming to avoid inconsistencies in radiological reporting in the future.

### *SSL 2.3*

**Before fifty: imaging findings in early age-onset colorectal cancer**

A. Perez, D. Kim, P.J. Pickhardt; Madison, WI/US

**Purpose:** Colorectal cancer (CRC) incidence has increased in younger cohorts. Our purpose was to compare the imaging findings of early age-onset (EOA) CRC diagnosed at age <50 with traditional ages (≥50).
**Material and methods:** 50 consecutive EOA patients (mean age±SD: 43±6; 27F/23M; n=10, age <40) from 2007 to 2012 were identified from EHR/PACS query. Control group consisted of consecutive CRC patients presenting ≥50 age (n=50; mean age±SD: 68±13; 27F/23M). Various imaging features were assessed at CT (i.e., tumor visualization, location, length, attenuation, lymph node size, number, confidence). Demographics, clinical history, and overall survival were recorded.
**Results:** EAO CRCs were more distal (rectal) in location [54%, 26/50 vs control 22%, 11/50; p<0.001] with increased presentation with hematochezia (60%, 29/48 vs 21%, 10/48; p<0.001). No difference in visualization of primary tumor at CT (66%, 33/50 EOA vs 50%, 25/50 control; p=0.11). Visualized EAO tumors were longer (4.8±2.4 vs 3.5±1.5 cm; p=0.005), otherwise without significant imaging differences from controls. Regional lymphadenopathy (LAN) was common with EAO. 50% (25/50) of EOA cohort with lymph node ≥ 9mm in size, 22% (11/50) with <9mm abnormal lymph nodes (with morphologic changes), 28% (14/50) without LAN (vs controls 32%, 40%, 28%, respectively; p=0.100). No difference in overall survival. 21% (10/47) EAO subjects expired <2 years of diagnosis, compared to 31% (15/48) of controls (p=0.269) despite more advanced presentation (EOA stage 3 or 4 76% 38/50 versus 56% 27/49; p=0.034).
**Conclusion:** EAO CRC often presents with a rectal location and adenopathy at CT which may hold implications in imaging interpretation of young persons where CRC is not suspected.

### SSL 2.4

### The PROSPECT trial: improving the prediction of disease recurrence in primary colorectal cancer

V. Goh[1], S. Mallett[1], D. Hill[2], M. Rodriguez-Justo[1], D. Patel[1], S. Khan[1], R. Glynne-Jones[3], V. Boulter[3], S.A. Taylor[1], S. Halligan[1]; [1]London/UK, [2]Birmingham/UK, [3]Northwood/UK

**Purpose:** Despite current best treatment, up to half of the colorectal cancer patients will develop recurrent disease by 5 years. Better identification of at-risk patients is an unmet need. We aimed to develop a prognostic model for disease recurrence via prospective evaluation of clinical, novel imaging and pathology variables.
**Material and methods:** Adult participants with primary colorectal cancer were recruited prospectively from 13 hospitals for this prognostic study with 36-month follow-up (ISRCTN 95037585). Exclusions were metastatic disease, prior cancer(s), and contraindications to contrast agents. In addition to standard staging and pathology, participants underwent perfusion CT, immunohistochemistry for angiogenesis and hypoxia, and mutation status analysis. A series of time-to-event nested prognostic models were developed and compared to current staging and each other. Sensitivity and specificity for recurrence based on Nelson–Aalen estimates at 3 years were calculated.
**Results:** 326/448 enrolled participants (226 male; median 67 years (range: 28–92) comprised the final cohort. 52 participants developed metastases; 12 had new cancers; 17 died as the first event. With 'high-risk' patients defined by standard practice—node-positive (Stage III) disease—sensitivity was 0.56 [95% CI: 0.44, 0.67], while specificity was 0.58 [95%CI: 0.51,0.64] for recurrence. Sensitivity and specificity for the best model (including perfusion CT variables) were 0.59 [95% CI: 0.46, 0.70] and 0.75 [95% CI: 0.68, 0.81], respectively, showing improved specificity.
**Conclusion:** Prognostication is improved by the inclusion of tumour stage, age, treatment group, and the presence of extramural vascular invasion. Novel imaging and pathology did not further increase model performance substantially.

### SSL 2.5

### Central versus local review of perfusion CT imaging variables from the PROSPECT trial of colorectal cancer

D. Prezzi[1], V. Goh[1], S. Mallett[2], D. Hill[3], M. Rodriguez-Justo[1], D. Patel[1], S. Khan[1], R. Glynne-Jones[4], V. Boulter[4], S.A. Taylor[1], S. Halligan[1]; [1]London/UK, [2]Prestwood/UK, [3]Birmingham/UK, [4]Northwood/UK

**Purpose:** Perfusion CT imaging relies on analysis software, linked to the scanner acquisition, to provide vascular measurements of the tissue of interest. In a multicentre setting, different scanners, acquisition/reconstruction parameters, software/mathematical analysis models and/or readers potentially contribute to measurement variability. We assessed the variability of perfusion CT measurements in the PROSPECT trial.
**Material and methods:** Adult participants with suspected or proven primary colorectal cancer were recruited prospectively from 13 hospitals (ISRCTN 95037585). Exclusions were metastatic disease at staging, prior cancer(s), and contraindications to contrast agents. Perfusion CT at local sites was analysed by 25 radiologists using software based on deconvolution, distributed parameter, slope or Patlak analysis (GE, Siemens, Phillips or Toshiba). Imaging was reviewed centrally by 3 experienced radiologists. Bland–Altman methods determined limits of agreement between local and central review measurements. Potential sources of variation including scanner and region of interest were assessed graphically.
**Results:** 291/303 (96%) participants imaged successfully were included in this analysis as technical issues precluded central review in 14/303 (4%). Mean difference [95% limits of agreement] for blood flow (mL/min/100mL), blood volume (mL/100mL), and permeability surface area product (mL/min/100mL) were -9.5 [-71.2, +52.3], -2.6 [-13.4, +8.19], and 3.1 [-27, +33.1], respectively. Plotted data indicated differences in the region of interest between local and central reviews reflecting observer variation, but this was not a major contributor to variance. Similarly, scanner type did not impact substantially the variance.
**Conclusion:** Variation in derived measurements, particularly vascular permeability, highlights ongoing challenges for quantitative imaging in a multicentre setting.

### SSL 2.6

### Assessment of lymph node status and tumor response after chemoradiation therapy in locally advanced rectal cancer: comparison of three methods of region of interest for intravoxel incoherent motion parameters

Y. Yuan, H. Pu, X.-L. Chen, H. Li; Chengdu/CN

**Purpose:** To assess the diagnostic performance of region of interest (ROI) methods of intravoxel incoherent motion diffusion-weighted imaging (IVIM-DWI) for determining lymph node metastases (LNM) and tumor response after chemoradiation therapy (CRT) in locally advanced rectal cancer (LARC).
**Material and methods:** 79 patients underwent preoperative IVIM-DWI before and after CRT. IVIM-DWI parameters apparent diffusion coefficient (ADC), slow diffusion coefficient (D), fast diffusion coefficient (D*), perfusion-related diffusion fraction (f) and their percentage changes [Δ%] were obtained according to three ROI protocols: whole volume, single slice and small samples. Risk factors were evaluated through logistic regression analyses. Areas under the receiver operating characteristic curves (AUCs) were calculated to evaluate diagnostic performance. Disease-free survival was estimated using Kaplan–Meier survival curves.
**Results:** Interobserver agreement was good for pre- and post-CRT whole-volume ROI and single-slice ROI (intraclass correlation coefficient [ICC], 0.775–0.953), and moderate for small samples ROI (ICC, 0.581–0.905). As for LNM, pCR and good response, AUCs for whole-volume ROI-derived Δ%D were higher than that of the other IVIM-DWI parameters (AUC, 0.810, 0.851, 0.903 vs. 0.609–0.790). In multivariate analysis, whole-volume ROI-derived Δ%D was an independent risk factor for discriminating LNM, pCR and good response (odds ratio, 0.947, 0.952, 0.805; p=0.001, <0.001, <0.001). After CRT, patients with LNM or poor response showed earlier recurrence (hazard ratio, 3.408, 3.498; 95% confidence interval, 1.289–9.012, 1.018–12.021; p=0.013, 0.047, respectively).
**Conclusion:** Whole-volume ROI-derived Δ%D provided high diagnostic performance and was an independent factor for evaluating LNM, pCR and good response. Furthermore, patients with LNM or poor response may have worse outcomes regarding recurrence.

### SSL 2.7

### Vascular but not metabolic phenotype is associated with neoadjuvant therapy response in primary esophageal/esophagogastric cancer

S.J. Withey[1], K. Owczarczyk[1], M. Grzeda[1], C. Yip[2], R. Neji[3], A. Green[1], J. Bell[1], H. Deere[1], M. Green[1], G. Cook[1], V. Goh[1]; [1]London/UK, [2]Singapore/SG, [3]Frimley/UK

**Purpose:** The current standard-of-care for potentially curable esophageal/esophagogastric cancer is neoadjuvant therapy prior to surgery. Imaging biomarkers that can predict response to neoadjuvant treatment may help to deliver individualized care. We aimed to investigate whether the tumor vascular or metabolic phenotype, assessed with dynamic contrast-enhanced MRI (DCE-MRI) and 18F-fluorodeoxyglucose positron emission tomography/CT (18F-FDG PET/CT), provides predictive and/or prognostic information beyond current staging.
**Material and methods:** Following ethical approval, participants with potentially resectable esophageal/esophagogastric cancer underwent DCE-MRI and 18F-FDG PET/CT prior to neoadjuvant therapy. Vascular ($K^{trans}$, $v_e$, $k_{ep}$, peak enhancement integral (PEI)) and metabolic ($SUV_{max}$, $SUV_{mean}$, metabolic tumour volume, total lesion glycolysis) parameters were generated from MRI and PET, respectively, and compared with a pathological response (Mandard tumour regression grade (TRG)) and recurrence-free or overall survival using logistic regression modelling.
**Results:** 39 participants (30 male; median 65 years, range: 54–72) underwent successful imaging. In multivariable analysis (adjusted for age, gender, T/N stage), lower PEI and higher $K^{trans}$ were potentially predictive of response to neoadjuvant therapy (TRG 1-2). For PEI, odds of response decreased by 5% for each 0.010 increase in PEI (OR = 0.95; 95% CI 0.90-1.00; p=0.03); for $K^{trans}$, odds of response increased by 13% for 0.010 increase in $K^{trans}$ (OR = 1.13; 95% CI 1.00-1.28; p=0.05). PET parameters were not predictive of response. No relationships between any imaging parameters and recurrence-free or overall survival were identified.
**Conclusion:** DCE-MRI biomarkers of perfusion and neoangiogenesis are predictive of response to neoadjuvant therapy in primary esophageal/esophagogastric carcinoma.

**13:15 - 14:15** **CHANNEL 3**

*Scientific Session Live SSL 3*

### Best papers in GI luminal and liver imaging

### SSL 3.1

### Contribution of standard MR enterography protocol sequences to diagnostic accuracy in Crohn's disease: results from the METRIC trial

G. Bhatnagar[1], S.A. Taylor[2], S. Mallett[2], L. Quinn[3], R. Beable[4], R. Greenhalgh[5], A. Higginson[4], R. Ilangovan[6], H. Lambie[7], E. Mainta[5], U. Patel[6], F. Porte[8], H. Sidhu[2], A. Slater[9], D. Tolan[7], I. Zealley[10], S. Halligan[2], T. Metric Trial Investigators[2]; [1]Frimley/UK, [2]London/UK, [3]Birmimgham/UK, [4]Portsmouth/UK, [5]Middlesex/UK, [6]Harrow/UK, [7]Leeds/UK, [8]Tunbridge Wells/UK, [9]Oxford/UK, [10]Dundee/UK

**Purpose:** To evaluate the diagnostic impact of diffusion-weighted images (DWI) and contrast-enhanced (CE) images in small bowel Crohn's disease.
**Material and methods:** Datasets from 73 patients (mean age 32, 40 male) (28 newly diagnosed, 45 relapsed patients) were each independently read by two radiologists selected from a pool of 20. Radiologists read the datasets in three sequence blocks: 1) T2 fast spin-echo (FSE) + fast imaging with steady-state free precession (FISP); 2) block one sequences + DWI; 3) block 2 sequences + CE images, documenting presence, location and activity of small bowel disease for each sequence block. Sensitivity and specificity (both readers combined) were calculated for disease presence and extent (i.e. full correct segmental location) against an outcome-based construct reference standard.
**Results:** 59/73 patients had small bowel disease. CE sequences were missing in 7. Per patient sensitivity for disease detection was essentially identical for the three sequence blocks (80% [95% CI 72, 86], 81% [73, 87], and 79% [71, 86], respectively). Specificity was identical (82% [64 to 92]). Per patient sensitivity for disease extent was 56% (47, 65), 56% (47, 65) and 52% (43 to 61), respectively, and specificity was 82% (64 to 92) for all sequence blocks. Sensitivity for active disease was 97% (90, 99), 97% (90, 99) and 98% (92, 99) and specificity was 8 (2, 35), 33 (14, 61) and 18 (5, 48), respectively. Results were consistent across segments and newly diagnosed/relapse patients.
**Conclusion:** There is no additional diagnostic benefit of adding either DWI or CE sequences to a basic T2 FSE and FISP combination suggesting MRE protocols can be safely simplified.

### SSL 3.2

### Fibrosis and disease activity (MAGNIFI-CD index) at MRI of Crohn's perianal fistulas in relation to long-term clinical closure

K. Van Rijn, E. Meima-Van Praag, P.M. Bossuyt, K. Horsthuis, H.J. Snijder, J. Tielbeek, C. Buskens, J. Stoker; Amsterdam/NL

**Purpose:** To evaluate differences in fibrosis and disease activity (MAGNIFI-CD index) at MRI of Crohn's perianal fistulas in relation to long-term clinical closure in patients treated with anti-tumor necrosis factor (TNF) and/or surgery.
**Material and methods:** Perianal Crohn's patients who received anti-TNF and/or surgery as part of previous studies between 2013 and 2020 and had a follow-up (FU)-MRI were retrospectively included. A radiologist scored the degree of fibrosis and the MAGNIFI-CD index at the FU-MRI. Long-term clinical closure was determined 12 months after the FU-MRI and was defined as no production on palpation and/or a closed external opening and absence of re-interventions. MRI findings were compared between patients with long-term clinical closure and those without.
**Results:** Fifty-two patients were included: 32 female, median age 33 years (IQR 26–46). FU-MRI was performed at a median of 9 months (IQR 6–14) after treatment. In 41/52 patients, long-term clinical FU-data were available, 26 patients showed clinical closure and 15 did not. At FU-MRI, ≥80% fibrosis was seen in 15 patients with and two patients without long-term clinical closure; for <80% fibrosis, this was 11 and 13, respectively. The median MAGNIFI-CD index was 0 (IQR 0–5) in patients with long-term clinical closure and 16 (IQR 9–20) in patients without (p=0.002*).
**Conclusion:** A high degree of fibrosis at MRI of Crohn's perianal fistula seems associated with long-term clinical closure. Furthermore, the MAGNIFI-CD index is lower in patients with long-term clinical closure than in patients without. The degree of fibrosis and MAGNIFI-CD index might have a predictive value in determining long-term clinical closure of perianal fistulas.

### SSL 3.3

### Reliability of LI-RADS v.2018 and EASL criteria in small liver observations using extracellular and hepatobiliary contrast agents: a comparative study

M. Ronot[1], V. Sapena[2], G. Brancatelli[3], A. Paisant[4], M. Renzulli[5], S. Terraz[6], M. Wagner[7], J. Rimola[2]; [1]Clichy/FR, [2]Barcelona/ES, [3]Palermo/IT, [4]Angers/FR, [5]Bologna/IT, [6]Geneva/CH, [7]Paris/FR

**Purpose:** To assess the interreader agreement of LI-RADS v.2018 and EASL criteria for the diagnosis of HCC with extracellular agents (ECA) and hepato-biliary contrast agents (HBA)-enhanced MRI, and to determine the effect of ancillary features (AF) and reader expertise on LI-RADS categorization.
**Material and methods:** 92 observations <3cm explored with both ECA and HBA-MRI acquired at ≤1-month interval were reviewed by 9 independent readers. Representative images for each observation were presented in random order in a digital atlas. Readers assessed EASL criteria, LI-RADS v.2018 categories, Li-RADS major features +/-ancillary features (AF) (and subsequent re-categorization) for each observation. Intraclass correlation coefficients (ICC) were calculated for LI-RADS categorization and Cohen's kappa was assessed for major features and EASL criteria.
**Results:** Kappa and ICC for EASL, LI-RADS category and LI-RADS re-categorization with AF were 0.72 (95%CI 0.63–0.8), 0.67 (0.62–0.70), and 0.65 (0.59–0.69) for ECA-MRI, and 0.69 (0.60–0.77), 0.67 (0.64–0.70) and 0.65 (0.61–0.68) for HBA-MRI, respectively. Kappa of ECA-MRI and HBA-MRI were 0.71 (0.42–1.0) and 0.68 (0.47–0.89) for non-rim arterial phase hyperenhancement, 0.5 (0.32–0.68) and 0.73 (0.61–0.85) for nonperipheral washout, and 0.57 (0.45–0.69) and 0.53 (0.40–0.66) for enhancing capsule. AF for LI-RADS categorization were applied in 17% (8.7–19.6%) and 28.3% (22.8–33.7%) of observations for ECA and HBA, respectively (p value=0.004). LI-RADS categorization adjusted by expertise was similar on both contrast agents 0.66 (0.62–0.7) and 0.67 (0.63–0.69) for ECA and HBA.
**Conclusion:** Inter-reader agreement for EASL and LI-RADS categorization was substantial. No appreciable differences were observed between contrast agent, AF application or reader expertise.

### SSL 3.4

### Liver volume: association with anthropometric parameters and liver function

A. Messner[1], I. Shchekoturov[2], D. Venidiktova[3], A. Kristic[1], M. Raudner[1], S. Pötter-Lang[1], N. Bastati-Huber[1], A. Ba-Ssalamah[1]; [1]Vienna/AT, [2]Moscow/RU, [3]Smolensk/RU

**Purpose:** Data on liver volume from 1315 patients were analyzed with respect to gender, age, body surface area (BSA), prevalence of cirrhosis, signs of portal hypertension and liver function parameters.
**Material and methods:** We analyzed liver volume, unenhanced T1 VIBE and gadoxetic acid-enhanced T1 VIBE, 20 minutes after injection (hepatobiliary phase, HBP), as well as T1 mapping before and after gadoxetic acid in the HBP and calculated liver function parameters (reduction rate of T1 relaxation time (rrT1), T1 relaxation velocity index (ΔR1), and relative liver enhancement (RLE)). Statistical analysis using the t test, Pearson correlation, and multiple stepwise linear regression was performed.
**Results:** The liver volume was significantly higher in male than in female patients ($p<0.001$), and in case of cirrhosis ($p=0.033$) and portal hypertension ($p<0.001$). A positive moderate correlation was found between BSA and liver volume ($r=0.497$, $p<0.001$). A weak negative correlation was found for age versus liver volume in a subgroup of subjects >55years of age, without cirrhosis and correcting for weight ($r=0.204$, $p<0.001$). Regression analysis revealed BSA, gender, age, prevalence of cirrhosis, and signs of portal hypertension to be predictors of liver volume (gender $p=0.021$, cirrhosis $p=0.006$, rest $p<0.001$). Weak, negative correlations were found between liver volume and liver function parameters (rrT1: $r= -0.226$, ΔR1: $r=-0.365$, RLE: $r=-0.235$; all $p<0.001$).
**Conclusion:** Anthropometric parameters seem to have a significant influence on liver volume, as do cirrhosis and portal hypertension. Age starts to play a role in the second half of life. Our data indicate that liver function parameters might decline with increasing liver volume.

### SSL 3.5

### Clinical and gadoxetic acid-enhanced MRI features associated with high-risk hepatic adenomas

J.R. Tse, B. Naini, D. Lu, S. Raman; Los Angeles, CA/US

**Purpose:** To identify clinical and quantitative gadoxetic acid (Gd-EOB-DTPA)-enhanced MRI features associated with high-risk hepatic adenomas (defined as beta-catenin-activated exon 3 genotype (BCAex3) or hemorrhage on histology).
**Material and methods:** In this retrospective, institutional review board-approved, and Health Insurance Portability and Accountability Act-compliant study, 64 hepatic adenomas (HCAs) from 53 adult patients (46 female, 7 male) from 2009 to 2020 with Gd-EOB-DTPA-enhanced MRI and tissue pathology were reviewed. HCAs were classified as inflammatory (I-HCA), hepatocyte nuclear factor-1 alpha mutated (H-HCA), beta-catenin-activated exon 3 (B-HCA), mixed beta-catenin-activated exon 3 inflammatory (BI-HCA), or unclassified (U-HCA) with immunohistochemistry. Signal intensity ratios (SIR) were calculated by comparing regions of interest of HCA to the adjacent liver.
**Results:** 64 HCAs were classified into 31 I-HCA, 16 H-HCA, 6 B-HCA, 4 BI-HCA, and 7 U-HCA. BCAex3 genotype was associated with male sex ($p=0.004$; logistic regression) but not symptoms, AFP, age, BMI, or number of HCAs. Increased HCA size was associated with both hemorrhage ($p=0.004$) and BCAex3 genotype ($p=0.001$). On Gd-EOB-DTPA-enhanced MRI, only the hepatobiliary phase could distinguish among subtypes. Hepatobiliary phase SIR ± standard deviation were I-HCA=0.70±0.08; H-HCA =0.46±0.12; B-HCA or BI-HCA=1.04±0.21; U-HCA=0.75±0.12; $p=0.004$, one-way ANOVA. Post hoc Tukey test for multiple comparisons showed that SI ratios for I-HCA, H-HCA, and B-HCA or BI-HCA were statistically different from each other ($p<0.0001$). Sensitivity and specificity of hepatobiliary phase SIR>0.8 for identifying BCAex3 genotype were 90% and 93%, respectively.
**Conclusion:** BCAex3 genotype was associated with higher hepatobiliary phase SIR, larger HCA size, and male sex. Histologic hemorrhage was associated with larger size irrespective of subtype. These features may help risk stratify HCAs noninvasively.

### SSL 3.6

### MRI-assessed enteric dysmotility and luminal content analysis in irritable bowel syndrome patients with constipation and bloating

R. Gollifer, S.A. Taylor, A. Menys, N. Zarate-Lopez, D. Chatoor, A. Emmanuel, D. Atkinson; London/UK

**Purpose:** Gastrointestinal symptoms in irritable bowel syndrome (IBS) occur without any discernible structural gut abnormality. Preliminary observations on MR enterography (MRE) suggest possible abnormal content and motility of the terminal ileum (TI) in constipation-predominant IBS (IBS-C) patients with bloating. We investigated whether MRI can quantify differences in small bowel (SB) content and motility between IBS-C patients and healthy controls (HCs).
**Material and methods:** 18 IBS-C patients with severe bloating (mean age 39, range 21–56 years; 17 female) and 20 HCs (mean age 28, range 22–48 years; 6 female) underwent MRE, including dynamic motility and anatomical sequences. Three texture analysis (TA) parameters assessed the homogeneity of the luminal content, with ratios calculated between the TI and 1) the SB and 2) the ascending colon. Four TI motility metrics were derived. Ascending colon diameter was measured. Comparison between HCs and IBS-C patients was performed independently for 1) the three TA parameters, 2) four TI motility metrics, and 3) ascending colon diameter.
**Results:** Compared to HCs, IBS-C patients had TI:colon ratios higher for TA contrast ($P < 0.001$), decreased TI motility [lower mean motility ($P = 0.04$), spatial motility variation ($P = 0.03$) and area of motile TI ($P = 0.03$)], and increased ascending colon diameter ($P = 0.001$).
**Conclusion:** IBS-C patients show reduced TI motility and differences in luminal content compared to healthy controls. This potentially indicates reflux of colonic contents or delayed clearance of the TI which alongside increased ascending colon diameter may contribute to symptoms of constipation and bloating.

### SSL 3.7

### Intestinal malrotation in adults: prevalence and findings based on CTC

A. Perez, P.J. Pickhardt; Madison, WI/US

**Purpose:** Intestinal malrotation, or nonrotation, is largely a pediatric diagnosis, but initial detection can be made in adulthood. CTC provides an ideal means for adult diagnosis and estimating prevalence. The purpose of this study was to evaluate the prevalence and imaging findings of intestinal malrotation in asymptomatic adults at CTC screening, as well as incomplete optical colonoscopy (OC) referral.
**Material and methods:** The CTC database of a single academic institution was searched for cases of intestinal malrotation. Prevalence was estimated from 9,844 adults undergoing initial CTC screening and 1,332 referred for incomplete OC. Demographic, clinical, and imaging data were reviewed.
**Results:** 27 cases of malrotation were confirmed (mean age, 62±9 years; 15M/12F), including 17 from the CTC screening cohort (0.17% prevalence) and 10 from incomplete OC (0.75% prevalence; $p<0.001$). Most cases (56%; 15/27) were initially diagnosed at CTC. In 67% (12/18), the presence of malrotation was missed on at least one relevant abdominal imaging examination. At least 22% (6/27) had a history of unexplained, chronic intermittent abdominal pain. At CTC, the SMA–SMV relationship was normal in only 11% (3/27). The ileocecal valve was located in the RLQ in only 22% (6/27). Two patients (7%) had associated findings of heterotaxy (polysplenia).
**Conclusion:** The prevalence of intestinal malrotation was over four times greater for patients referred from incomplete OC compared with primary screening CTC, likely related to anatomic challenges at endoscopy. Malrotation was frequently missed at other abdominal imaging examinations. CTC can uncover unexpected cases of malrotation in adults, which may be relevant in potential future complications.

ON DEMAND

*Scientific Session On Demand SSD 1*
### Imaging pancreatic disorders: new insights

### SSD 1.1
### Can preoperative CT scan and bioimpedance vector analysis help to predict the development of pancreatic fistula?
C. Maino[1], D. Ippolito[2], M. Ragusi[2], D. Gandola[2], C. Talei Franzesi[2], T.P. Giandola[2], S. Sironi[1]; [1]Milan/IT, [2]Monza/IT

**Purpose:** To evaluate the accuracy of the bioimpedance vector analysis (BIVA) in predicting pancreatic steatosis (PS) and development of postoperative pancreatic fistula (POPF), compared with preoperative CT scan and the pathologic specimen.
**Material and methods:** A total of 75 patients who underwent pancreatic resection having a preoperative CT staging for pancreatic cancer were prospectively enrolled. All CTs were analyzed to determine the overall mean attenuation value of the pancreas, excluding focal lesions, expressed as Hounsfield unit (HU). Moreover, a radiologist drew three different regions of interest (ROI), located in the head, body, and tail, to calculate the mean attenuation value at these levels. BIVA was performed the day before surgery and pancreatic steatosis was assessed with the fat mass index (FMI). Spearman correlation and ROC analysis were used to analyze and compare the techniques.
**Results:** The mean preoperative computed tomography pancreatic attenuation value was 18 (-3-39), and the mean FMI was 7.2 (3.4-11). Positive linear correlations were found between mean HU value and FMI when compared to histologic data (r= -0,852, p<0.001 and r= 0.652, p<0.001, respectively), and a good correlation was found between HU value and FMI (r= -0,659, p<0.001). All methods reported a good diagnostic accuracy in determining POPF, resulting in an AUC of 0.924 (95%CI: 0.844-1), 0.884 (95%CI: 0.778-0.990), and 0.942 (95%CI: 0.879-1) for mean HU value, FMI, and histology, respectively.
**Conclusion:** Preoperative fat mass evaluation through mean CT attenuation value and BIVA can be considered good predictors of POPF, and also a reliable approach to quantify pancreatic steatosis.

### SSD 1.2
### Malignancy prediction in patients with intraductal papillary mucinous neoplasm: are we getting better with additional new imaging features?
R. Pozzi Mucelli[1], C.F. Moro[1], R. Valente[2], M. Del Chiaro[3], M. Löhr[1], N. Kartalis[1]; [1]Stockholm/SE, [2]Umeå/SE, [3]Aurora, CO/US

**Purpose:** Current guidelines base the management of intraductal papillary mucinous neoplasm (IPMN) on several resection criteria (RC). However, most of the previously published studies evaluating RC include also patients with solid pancreatic cancer (PC) at preoperative imaging, which may affect RC yield. The purpose of this study is to evaluate if currently suggested RC are able to predict high-grade dysplasia/invasive cancer (HGD/INV) in branch-duct (BD) and mixed-type IPMN without solid PC, and whether new features (cyst volume, diameter ratio, uni/multifocality) may be associated with a higher risk of HGD/INV.
**Material and methods:** This is a retrospective cohort study, including 106 patients with histopathological diagnosis of BD- and mixed-type IPMN, and preoperative MRI. Patients with solid PC were excluded. Imaging and clinical features were collected, and cyst volume was calculated on T2-w images. Logistic regression analysis was performed and predicted probabilities (PP) were calculated. Statistical significance was set at two-tailed p<.05.
**Results:** Contrast-enhancing mural nodules (MN), main pancreatic duct (MPD)≥5mm and CA19-9 (>37μ/ml) were the only parameters associated with higher risk of HDG/INV at uni- and multivariable logistic regression [MN OR:4.33, 95%CI: 1.18–15.75, p=0.02; MPD≥5mm OR:4.2, 95%CI: 1.34–13.1, p=0.01; CA19-9(>37μ/ml) OR:6.72; 95%CI: 1.89–23.89, p=0.003]. The PP for HGD/INV was 0.05 (95%CI: 0.01–0.15) with none of parameters, 0.18 (95%CI: 0.05–0.5) with MN, 0.49 (95%CI: 0.2–0.78) by further addition of the factor MPD≥5mm and increased to 0.87 (95%CI: 0.55–0.97) with the presence of all three parameters.
**Conclusion:** MN, dilated MPD and elevated CA19-9 were associated with HGD/INV in patients with BD- and mixed-type IPMN without preoperative solid PC. Additional new features (cyst volume, diameter ratio, uni-/multifocality) failed to improve the prediction of HGD/INV preoperatively.

### SSD 1.3
### Quantitative edge analysis of pancreatic margins in patients with chronic pancreatitis: a correlation with exocrine function
A. Grecchi, M.C. Ambrosetti, A. Ambrosetti, A. Amodio, G.A. Zamboni, G. Mansueto; Verona/IT

**Purpose:** To correlate a computer-aided analysis of pancreatic margins with exocrine function measured with fecal elastase values in patients with chronic pancreatitis (CP).
**Material and methods:** The hospital registries were searched for patients with confirmed diagnoses of CP with fecal elastase values and abdomen MRI performed in our institute in less than 1 year. We identified 123 patients divided into 3 groups of 41 patients each based on the fecal elastase value: group A with fecal elastase <100 μg/g; group B with fecal elastase between 100 and 200 μg/g; group C with fecal elastase > 200 μg/g. An in-house software was developed, and computer-assisted quantitative edge analysis was performed on pancreatic margin non-contrast-enhanced water-only Dixon T1-weighted images of the pancreas with chronic inflammation changes, obtaining the root mean square deviation $S_D$ of the actual border from the average boundary line. Border $S_D$ values were compared across groups using a Kruskal–Wallis test and correlation between border $S_D$ values and fecal elastase values were tested with the Spearman test.
**Results:** A significant difference in SD values was observed between the three groups (p<0.0001). A significant correlation was observed between SD values and elastase values with $\rho$ =0.5945.
**Conclusion:** Quantitative edge analysis with dedicated software may stratify patients with chronic pancreatitis according to the degree of exocrine insufficiency, potentially contributing to the morphological and functional staging of this pathology.

### SSD 1.4

### An abbreviated MRI protocol for surveillance of cystic pancreatic lesions

F. Delaney, H. Fenlon, C. Cronin; Dublin/IE

**Purpose:** The appropriate imaging surveillance strategy for lower risk cystic pancreatic lesions (CPLs) (branch duct—intraductal papillary mucosal neoplasms and indeterminant small cystic lesions) has been a topic of intense study in recent years. CPL surveillance currently creates a significant burden on MRI resources as follow-up studies are recommended every 6–24 months over a prolonged period and a standard protocol pancreatic MRI acquisition time may be up to 50 minutes. Recently, abbreviated MRI (A-MRI) protocols for specific clinical scenarios have been successfully implemented in other areas and the necessity of contrast-enhanced and diffusion-weighted imaging (DWI) for routine follow-up of CPLs has been questioned.
**Material and methods:** We reviewed the current literature using an evidence-based approach to determine whether an A-MRI protocol for CPL surveillance may be safely adopted.
**Results:** A-MRI studies help avoid the routine use of comprehensive protocols with lengthy acquisition times and potentially unnecessary gadolinium administration. A number of recent retrospective studies have indicated that an A-MRI, omitting contrast-enhanced and DWI, may be used for CPL surveillance without any suspicious features, as defined in current clinical guidelines, or cases of malignancy being missed indicating an acceptable negative predictive value for surveillance imaging. A small number of cases may need to be recalled for additional MR sequences based on the A-MRI findings; however, there is still a significant overall timesaving.
**Conclusion:** Best available evidence currently suggests that an A-MRI protocol should be considered for routine CPL surveillance. This may be reflected in future clinical guidelines for CPL management which do not advise A-MRI protocols at present.

### SSD 1.5

### How are imaging findings associated with exocrine insufficiency in idiopathic chronic pancreatitis?

A. Chandramohan, R. Shetty, G. Kumbhar, A. Thomas, S.D. Chowdhury; Vellore/IN

**Purpose:** To study the association between imaging findings in chronic pancreatitis and faecal elastase (FE1) in patients with idiopathic chronic pancreatitis (ICP).
**Material and methods:** In this retrospective study on prospectively maintained database of patients with ICP, a radiologist blinded to clinical and laboratory findings reviewed CT and/or MRI. Findings were documented according to recommendations of the Consortium for the Study of Chronic Pancreatitis, Diabetes, and Pancreatic Cancer, October 2018. Pancreatic exocrine insufficiency was assessed by FE1 test. Association between imaging findings and FE1 was studied using chi-square test or independent sample t test.
**Results:** 70 patients (M:F= 37:33) with ICP with a mean age of 24.2 (SD6.5) years (range 10–37 years) and mean disease duration of 5.6 (SD 4.6) years (range 0–20 years) were included. Mean FE1 level was 82.5 (SD120.1) (range 5–501) µg elastase/g. Mean main pancreatic duct (MPD) calibre was 7(SD4) mm (range 3–21 mm) and mean pancreatic parenchymal thickness (PPT) was 13.7 (SD5.5) mm (range 5–27 mm). Low FE1 (<100) was associated with MPD size, PPT, type of pancreatic calcification; presence of intraductal stones, side branch dilatation on MRCP and extent of pancreatic involvement ($p<0.05$). 79%, 86% and 78% with moderate to severe MPD dilatation, pancreatic atrophy and side branch dilatation had low FE1, respectively. But nearly half of those with no or mild abnormality had low FE1.
**Conclusion:** Significant association between FE and imaging findings demonstrates its potential as a marker of exocrine insufficiency and disease severity in chronic pancreatitis. But imaging and FE are complementary rather than supplementary.

### SSD 1.6

### Pancreatic CT attenuation index as a marker of impaired glucose metabolism

A. Jeyakumar, P. Panneerselvam, R. Ramachandran, V.S. P.M.; Chennai/IN

**Purpose:** To assess the relationship between CT attenuation indexes and impaired glucose metabolism in a clinical setting.
**Material and methods:** This retrospective study was done from October 2019 to March 2020; 80 patients (47 men and 33 women; age range 35–60 years) who underwent CT were included in the study. Attenuation was measured in three regions of interest in the pancreas and the spleen on nonenhanced CT images. The difference between pancreatic and splenic attenuation and the pancreas-to-spleen attenuation ratio were calculated. A multivariate logistic regression model was used to determine whether CT attenuation indexes correlated with impaired glucose metabolism (i.e., impaired glucose tolerance, impaired fasting glucose, or presence of diabetes).
**Results:** Patient with impaired glucose metabolism was significantly correlated with the difference between pancreatic and splenic attenuation ($r = –0.621$, $P < .01$) and the pancreas-to-spleen attenuation ratio ($r = –0.611$, $P < .01$). The CT attenuation indexes were significant and independent variables predictive of impaired glucose metabolism.
**Conclusion:** Pancreatic CT attenuation indexes that are applied to the quantification of pancreatic fat are significantly associated with clinical assessment of impaired glucose metabolism. Accumulation of fat in the pancreas is the commonest cause of pancreatic dysfunction, leading to diabetes mellitus.

### SSD 1.7

### Assessment of pancreatic tumour response on LDE225, gemcitabine and nab-paclitaxel using intravoxel incoherent motion MRI

N. Wassenaar, E.N. Pijnappel, R. Klaassen, F. Struik, J. Stoker, J. Runge, H.W.M. Van Laarhoven, J.W. Wilmink, A.J. Nederveen, O.J. Gurney-Champion; Amsterdam/NL

**Purpose:** Survival of patients with pancreatic ductal adenocarcinoma (PDAC) remains poor despite the progress in treatment over the last decades. Stromal deposition in PDAC is thought to play a crucial role in preventing chemotherapy efficacy. In this study, the tumour and its stroma were targeted using chemotherapy (LDE225 and gemcitabine+nab-paclitaxel). The goal of this research was to assess PDAC's response with intravoxel incoherent motion (IVIM) MRI and to correlate IVIM parameters with overall survival (OS).
**Material and methods:** 34 patients with metastatic PDAC underwent a baseline MRI scan, of whom 21 underwent an additional scan after 8 weeks of treatment. Diffusion-weighted imaging (DWI) was performed at 3.0T using single-shot 2D echo-planar imaging, TR/TE:2145/46ms and 12 b-values (0–600). An IVIM model was fitted using bi-exponential fitting, obtaining diffusion (D), pseudodiffusion (D*) and perfusion fraction (f) maps. Mean values inside the region of interest were used to determine the overall effect of chemotherapy on the tumour, the correlation with OS and the predictive value of these parameters.
**Results:** A significant increase in D was seen during chemotherapy. Furthermore, significant correlations between OS and baseline f were found. The area under the receiver operating characteristic curve (AUC) was the highest for baseline f (AUC=0.835, sensitivity=93%, specificity=77%) to distinguish long (>220 days) from short OS.
**Conclusion:** The increase in D during treatment can be explained by necrosis and a decrease in stroma. Lower cellularity leads to higher D. A positive correlation was found between OS and the change in perfusion. IVIM MRI can be used for the evaluation of chemotherapy response on PDAC micro-environment.

### SSD 1.8

### Quantitative edge analysis for the diagnosis of pancreatic carcinoma

M. Bariani, G.A. Zamboni, M.C. Ambrosetti, A. Ambrosetti, G. Malleo, G. Mansueto; Verona/IT

**Purpose:** To assess with a quantitative technique the pancreatic border and differentiate normal and pathological findings.
**Material and methods:** From all patients who underwent MDCT for staging of pancreatic adenocarcinoma from January 2018 to January 2020, we selected 50 patients (36 males, 14 females; mean age 63.7 years) with adenocarcinoma of the body–tail of the pancreas. Computer-assisted quantitative edge analysis was performed on border fragments in MDCT images of neoplastic and healthy glandular parenchyma, obtaining the mean square deviation (SD) of the actual border from the average boundary line. SD values relative to healthy and neoplastic borders were compared by paired t-test.
**Results:** Tumoral border portions were systematically characterized by significantly lower SD values than healthy fragments (0.50 vs 1.27; p<0.001). In our series, using a threshold value of SD > 0.6855 pix showed 96% sensitivity and 96 % specificity in differentiating cancerous from healthy pancreatic edge segments.
**Conclusion:** We introduce a quantitative measure of boundary irregularity which clearly correlates with the presence/absence of pancreatic adenocarcinoma. Quantitative edge analysis can be promptly performed on select border fragments in MDCT images, thereby providing a useful supporting tool for diagnostics, and a possible starting point for machine-learning recognition based on lower dimensional feature space.

### SSD 1.9

### Efficacy of MRI in the non-invasive differentiation between focal pancreatitis and pancreatic cancer

A. Kristic, A. Messner, C. Spick, A. Herold, N. Bastati-Huber, S. Pötter-Lang, A. Ba-Ssalamah; Vienna/AT

**Purpose:** The purpose was to assess the diagnostic performance of MRI to non-invasively differentiate between focal pancreatitis and pancreatic cancer.
**Material and methods:** Dynamic contrast-enhanced MRI images, including DWI images and secretin-enhanced MRCP, were obtained. All patients were examined on a 3 Tesla unit. We evaluated the exams retrospectively and compared the imaging diagnosis with histopathology as the gold standard. The statistical analyses were performed using descriptive statistics, including the Pearson Chi-square test, the Fisher's exact test, as well as the Student's t-test, and logistic regression.
**Results:** A total of 96 subjects were included in our study. Forty-three participants had a benign pathology on histology, and 53 were malignant. MRI showed an accuracy of 85.42%, a sensitivity of 94.34%, and a specificity of 74.42%. Malignant lesions more often showed an inhomogeneous signal intensity in the arterial, equilibrium, and late phase, as well as a hyperintense appearance on DWI, with b values of 50, 300, and 600 sec/mm$^2$. In addition, vascular infiltration, ductal stenosis, and larger duct widths were observed. The "duct-penetrating sign," however, was observed in benign lesions.
**Conclusion:** MRI, with a special emphasis on DWI and a secretin-enhanced MRCP sequence, is an important tool in the diagnostic workup to distinguish between benign and malignant pancreatic tumors.

### SSD 1.10

### Role of contrast-enhanced US in grading the severity of acute pancreatitis

S. Kiran, R. Ananthakrishnan, G.S. Sreenath, R. Kashyap; Puducherry/IN

**Purpose:** To assess the diagnostic accuracy of contrast-enhanced US (CEUS) in grading the severity of acute pancreatitis and to correlate CEUS findings with clinical outcome variables such as hospital stay, need for intervention and BISAP score.
**Material and methods:** 56 patients with acute pancreatitis referred for CECT between January 2019 and August 2020 were included in the study, and B-mode USG and CEUS were performed in all these patients. Parameters such as pancreatic size and enhancement, peripancreatic fluid collections and extrapancreatic complications were recorded in CEUS and compared with CECT. Ultrasound severity index (USSI) and modified ultrasound severity index were calculated for each patient and compared with CT severity index and modified CT severity index, respectively.
**Results:** The sensitivity and specificity of CEUS in differentiating acute interstitial pancreatitis from acute necrotizing pancreatitis were 93.1% and 96.3%. The sensitivity of CEUS in diagnosing splenic vein thrombosis and peripancreatic fluid collections was 87.9% and 76.9%, whereas the specificity was 100% in both. The agreement between USSI and CTSI was calculated as 0.86 (Cohen's kappa coefficient) and between modified USSI and modified CTSI as 0.85, indicating an almost perfect agreement. No significant differences were noted between USSI and modified USSI in grading the severity of acute pancreatitis and both indices showed a good correlation with clinical outcome variables.
**Conclusion:** CEUS has a good diagnostic accuracy to detect necrosis and grade the severity of acute pancreatitis and can be used as an alternative in patients where CECT is contraindicated. USSI and modified USSI are equally good indicators to predict clinical outcome variables.

### SSD 1.11

### Optimization of MR elastography in the pancreas

A.-S. Van Schelt[1], N. Wassenaar[1], E. Schrauben[1], M. Troelstra[1], J.L. Nelissen[1], R. Sinkus[2], J. Stoker[1], A.J. Nederveen[1], J. Runge[1]; [1]Amsterdam/NL, [2]London/UK

**Purpose:** Pancreatic MR elastography (MRE) could allow for non-invasive estimation of tissue viscoelastic properties and has the potential to better characterize pancreatic inflammation and cancer. However, it remains challenging and requires careful optimization. We aimed to optimize a rapid interleaved multi-slice (Ristretto) gradient echo (GRE) acquisition for pancreatic MRE.
**Material and methods:** Acquisitions were performed with informed consent in 10 healthy volunteers at 3.0T using a gravitational transducer at 3x3x3mm$^3$ voxel-size (FOV: 384x256x27mm), SENSE2 and four consecutive breath-holds. Vibration frequency (40/50Hz), number of measured wave-phase offsets (4/5), and TE (6.9/9.2ms) were adjusted for optimization. Post-processing was performed using dedicated software. Analysis was performed on shear-wave amplitudes (SWA), deviation from a sinusoid (nonlinearity) and shear-wave speed (SWS) by averaging manually drawn regions-of-interest in the pancreatic head. Repeated measures analysis of variance and pairwise comparison were used to determine intra-subject variability for all quality parameters.
**Results:** The SWA was significantly higher at 40Hz than at 50Hz (40Hz=90+/-28µm; 50Hz=65+/-20µm; F(3,27)=11, p=0.00). Nonlinearities were acceptable for all scans (<50%), with no significant difference (F(3,27)=1.82, p=0.17). The average SWS for 40Hz scans was 0.97+/-0.5m/s (F(2,18)=1.64, p=0.22) and 1.17+/-0.04m/s for 50Hz.
**Conclusion:** The average pancreatic SWS was in line with the literature. The SWA significantly improved using 40Hz, deeming it the preferred choice. Measuring more offsets theoretically improves accuracy, though this was not evident in our limited study. Increasing TE can allow for measuring smaller amplitudes, although it lengthens breath-holds significantly. GRE-MRE at 40Hz, TE6.9ms and 4/5 wave-phase offsets can estimate the visco-elastic properties of the pancreas head and has acceptable breath-hold durations (<17s) for clinical purposes.

ON DEMAND

### *Scientific Session On Demand SSD 2*
### Focal Liver lesions

### *SSD 2.1*
**Outcomes of indeterminate LI-RADS observations occurring after hepatitis C virus eradication with direct-acting antiviral therapy**

R. Cannella, G. Pilato, F. Vernuccio, C. Celsa, C. Cammà, G. Brancatelli; Palermo/IT

**Purpose:** To assess the outcome of new indeterminate observations with low (LR-2), intermediate (LR-3), or high (LR-4) probability for HCC occurring in cirrhotic patients after direct-acting antiviral (DAA) therapy.
**Material and methods:** This retrospective study included consecutive hepatitis C virus-cirrhotic patients who obtained sustained virologic response after DAA therapy and with at least one LI-RADS indeterminate observation imaged with CT or MRI, not present at prior cross-sectional exams or newly detected observation on US screening. Two readers reviewed CT/MRI exams to categorize observations according to the LI-RADSv2018 and assess the evolution on subsequent CT/MRI follow-ups. Clinical and imaging predictors of evolutions were assessed by Cox proportional hazard model. Cumulative incidence of progression to malignant categories (LR-5 or LR-M) was assessed by Kaplan–Meier method.
**Results:** Fifty-four DAA-treated cirrhotic patients (median age 73.0 years, 72.2% males) with 80 indeterminate observations (10 LR-2, 57 LR-3, and 13 LR-4) were included. During a median follow-up time of 15 months (IQR 8-23 months), 26 (32.2%) and 2 (2.5%) observations progressed to LR-5 or LR-M categories, respectively. Overall, cumulative incidence of progression to malignant categories was 18.5% at six months, 20.0% at one year, and 22.5% at two years. Cumulative incidence was significantly higher in LR-4 vs LR-3 (log-rank P<0.001) and LR-4 vs LR-2 (log-rank P=0.001). Initial LI-RADS category (hazard ratio 4.16, 95% confidence interval 2.04-8.50, P<0.001) was the only independent predictor of progression to malignant categories by multivariate Cox analysis.
**Conclusion:** Initial LI-RADS category is an independent predictor of progression to malignancy categories in indeterminate observations occurring after DAA therapy.

### *SSD 2.2*
**Outcome of LR-3 and LR-4 observations without arterial phase hyperenhancement at gadoxetic acid-enhanced MRI follow-up**

I. Viola, F. Agnello, L. Rabiolo, F. Midiri, L. La Grutta, M. Galia; Palermo/IT

**Purpose:** The aim is to retrospectively evaluate the outcome of LR-3 and LR-4 observations without arterial phase hyperenhancement (APHE) and identify which features could predict LR-5 progression at gadoxetic acid (Gd-EOB-DTPA)-enhanced MRI follow-up.
**Material and methods:** 49 cirrhotic patients (55 LR-3 and 19 LR-4) without APHE were enrolled. LR-3 and LR-4 were classified as decreased, stable or increased in category at follow-up. The presence or absence of major and ancillary LI-RADS features, LI-RADS category and observation size was evaluated. Chi-square and Fisher's exact test were used to assess if baseline LI-RADS features and diameter (<10/ ≥10 mm) were associated with LR-5 progression of LR-3 and LR-4. P < .05 was considered statistically significant.
**Results:** Of 55 LR-3, 17 (31%) progressed to LR-5, 3 (6%) progressed to LR-4, 6 (11%) remained stable in category and 29 (52%) decreased in category. Of 19 LR-4, 8 (42%) progressed to LR-5, 4 (21%) remained stable in category and 7 (37%) decreased to LR-3. Major and ancillary features were not significantly different among LR-3 and LR-4 that progressed to LR-5 and those that remained stable or decreased in category. A diameter ≥ 10 mm significantly increased LR-5 progression risk of LR-3 (OR = 6.07; 95% CI: 0.12; 60.28]; P < .001); LR-4 with a diameter ≥ 10 mm more likely become LR-5 at follow-up (OR = 8.95; 95% CI: 0.73; 111.8; P = .083).
**Conclusion:** LR-3 and LR-4 without APHE were often downgraded or remained stable in category at MRI follow-up. LR-3 and LR-4 with a diameter ≥ 10 mm had an increased risk of LR-5 progression.

### *SSD 2.3*
**Influence of dilution on the transient severe motion artifacts and signal intensity of gadoxetic acid-enhanced liver MRI**

G.O. Dovjak, S.H. Polanec, S. Pötter-Lang, P. Baltzer, H. Bickel, A. Herold, A. Kristic, J.C. Hodge, M. Weber, N. Bastati-Huber, A. Ba-Ssalamah; Vienna/AT

**Purpose:** To investigate the effect of saline-diluted gadoxetic acid, done for arterial-phase (AP) artifact reduction, on signal intensity (SI), and hence focal lesion conspicuity on MRI.
**Material and methods:** We examined 118 patients who each had two serial gadoxetic acid-enhanced liver MRI performed at 1ml/sec, first with non-diluted, then with 1:1 saline-diluted contrast. Two blinded readers independently analyzed the artifacts and graded dynamic images using a 5-scale score. The absolute SI of liver parenchyma, focal liver lesions (if present), aorta, portal vein at the level of the celiac trunk, and paraspinal muscle, as well as the standard deviation of background noise, were measured in all phases. The normalized SI with muscle (SI_norm) and air signal-to-noise ratio (SNR) of these vascular structures and contrast-to-noise ratio (CNR) of focal liver lesions were calculated.
**Results:** AP artifacts were observed in 15 non-diluted (12.7%) and 6 diluted (5.1%) patients. A non-diagnostic score was assigned only in the non-diluted group in three patients (2.5%) and none in the diluted group. Both SI_norm and SNR values in the diluted group were significantly higher in the arterial phase for the aorta and hepatobiliary phase for the liver. The CNR was significantly higher for the lesion in the portal-venous and transitional phases. The inter-rater correlation coefficient was excellent (0.99).
**Conclusion:** Dilution of gadoxetic acid with saline 1:1, administered at an injection rate of 1ml/sec produces images with significantly fewer artifacts and equal or even higher SNR and CNR compared to standard non-diluted contrast.

### *SSD 2.4*
**Intact tumour capsule on contrast-enhanced CT as a prognostic marker for patients with HCC undergoing stereotactic body radiation therapy**

S.H. Mak, C.L. Chiang, S.M. Wong, K.W.H. Chiu; Hong Kong/HK

**Purpose:** Stereotactic body radiation therapy (SBRT) is a novel locoregional therapy for the treatment of HCC where intense radiation doses are delivered to the tumour. While promising, there is currently no radiological marker that can predict treatment response. In recent years, tumour capsule on cross-sectional imaging has been shown to be associated with improved prognosis and in this study, we aim to investigate the prognostic value of this radiological sign.
**Material and methods:** HCC patients that received SBRT in Tuen Mun Hospital between 2006 and 2017 were included for analysis if (i) tumour size ≥ 5cm, (ii) Child–Pugh score class A/B, and (iii) patient not eligible for curative treatment. The association between tumour capsule, treatment response and survival outcomes was evaluated.
**Results:** 156 patients, of which 84.6% were males, had a median age of 61 years (range: 29–85), were included for analysis. Intact tumour capsule (n=80; 58.4%) was associated with better SBRT response, as evaluated by RECIST [overall response rate (ORR) 62.5% vs 33.3%] (X2=15.1; P=0.001) and mRECIST (ORR 77.6% vs 38.6%) (X2=23.5; P<0.001) criteria. Intact tumour capsule was associated with progression-free survival [hazard ratio (HR) 0.68; 95% CI 0.48–0.96; p=0.03] and approach statistical significance for overall survival (HR 0.71; 95% CI 0.51–1.01; p=0.06). Median survival times for capsule positive and negative were 14.3 months (95% CI 10.22–18.45) and 11.0 months (95% CI 6.73–15.29), respectively.
**Conclusion:** Intact tumour capsule was associated with better response to SBRT and a trend of better survival. Further validation of findings in a larger cohort is warranted.

### SSD 2.5

### Accuracy of radiomics on gadoxetate disodium-enhanced MRI for the prediction of response in patients with HCC after transarterial embolization

R. Cannella, C. Cammà, F. Vernuccio, F. Matteini, C. Celsa, P. Giuffrida, A. Comelli, C. Cammà, G. Brancatelli; Palermo/IT

**Purpose:** To explore the potential of radiomics on gadoxetate disodium-enhanced MRI for the prediction of HCC response after transarterial embolization (TAE).

**Material and methods:** This retrospective study included cirrhotic patients undergoing the first single TAE between 2015 and 2020 with unifocal HCC naive to treatments. All patients underwent gadoxetate disodium-enhanced MRI with the same imaging protocol on 1.5-T MR scanners. Radiomics analysis was performed by two blinded readers using a dedicated research software. The whole lesion was segmented on portal venous, 3-minute transitional, and 20-minute hepatobiliary (HBP) phases. Post-TAE response, assessed according to mRECIST criteria, was used as the reference standard. Data were analyzed using the generalized linear model (GLM) regression and the discriminant analysis (DA). Areas under the receiver operating characteristic curve (AUROCs) were calculated for radiomics models.

**Results:** The final population included 51 patients (42 males, mean age 71.5±8.7 years). The mean size of HCCs was 23.7±11.3 mm. After TAE, complete response was obtained in 21 (41.1%) patients and partial response was obtained in 8 (15.7%) patients. Radiomics models incorporating four uncorrelated features showed the highest performances on HBP with an AUROC of 0.861 (95% CI 0.735–0.954, p=0.010), sensitivity of 75.4%, and specificity of 82.2% for the prediction of complete response, and an AUROC of 0.790 (95% 0.648–0.931, p=0.031), sensitivity of 58.7%, and specificity of 90.1% for the prediction of objective response (complete and partial responses).

**Conclusion:** Radiomics models based on gadoxetate disodium-enhanced MRI show good performance for the prediction of complete response in HCCs treated with TAE.

### SSD 2.6

### Intraindividual comparison of LI-RADS category and imaging features on contrast-enhanced CT and MRI

F. Agnello, R. Cannella, F. Vernuccio, M. Midiri, G. Brancatelli, M. Galia; Palermo/IT

**Purpose:** To perform an intraindividual comparison of LI-RADS category and imaging features in patients at high risk for HCC on contrast-enhanced CT and MRI.

**Material and methods:** This retrospective study included adult patients meeting the following criteria: 1) diagnosis of cirrhosis; 2) contrast-enhanced CT and MRI performed with a maximum interval of one month; 3) lack of interval treatment for target observations; 4) confirmation of benignity or malignancy by pathology or long-term follow-up. Two blinded radiologists evaluated the observations according to the LI-RADSv2018 diagnostic algorithm. Intraindividual differences were assessed using the McNemar test. Inter-modality agreement was calculated using the Cohen's kappa (k) test.

**Results:** A total of 73 observations (mean size 34.0 ± 32.4 mm) in 53 patients were included. There were no significant differences in major and imaging features between CT and gadoxetate disodium MRI (p≥0.063). Overall inter-modality agreement for LI-RADS categories was moderate (k value: 0.56, 95% CI 0.39–0.73). Inter-modality agreement between CT and Gd-EOB.DTPA MRI was moderate (k value of 0.52, 95% CI 0.32, 0.72) for all observations, while agreement was poor for the subset of observations ≤20 mm (k value -0.20, 95% CI -0.30, -0.09). Inter-modality agreement between CT and MRI with extracellular contrast was moderate (k value: 0.50, 95% CI 0.12–0.87). Agreement between Gd-EOB-DTPA and MRI with extracellular contrast was fair (k value: 0.26, 95% CI 0.06–0.47).

**Conclusion:** The inter-modality agreement for LI-RADS categorization is moderate between CT and MRI, but the agreement is low for observations ≤20 mm.

### SSD 2.7

### Synthetic diffusion-weighted MRI in patients with HCC: feasibility versus conventional acquired diffusion-weighted MRI

S. Yao, Y. Wei, B. Song; Chengdu/CN

**Purpose:** To investigate the clinical feasibility and diagnosis performance of synthetic diffusion-weighted imaging (DWI) in patients with HCC compared with conventional acquired DWI qualitatively and quantitatively.

**Material and methods:** Twenty-seven patients with HCC were prospectively involved undergoing conventional acquired DWI at b-values of 50, 800, 1000, 1200, and 1400 s/mm² and synthetic DWI, of which images were calculated at b=1000, 1200, and 1400 s/mm² from acquired images using b-values of 200 and 600 s/mm². Two readers reviewed image sets of both synthetic and conventional acquired images of 1000, 1200, and 1400 s/mm² independently and rated image quality (4-point scale) and lesion conspicuity (5-point scale). Quantitative analysis includes signal-to-noise ratio (SNR), contrast-to-noise ratio (CNR), SIR, and cancer-to-parenchyma contrast ratio. Statistical comparisons were performed using the Friedman test, analysis of variance, and intraclass correlation coefficient (ICC).

**Results:** No significant differences in overall image quality and lesion conspicuity at both synthetic and conventional acquired DWI sets (p>0.05) were found, whereas $synDWI_{1400}$ showed lower overall image quality than other DWI sets (p<0.001). SNR, CNR, and SIR showed a continuous decrease as b-values increased, with significant differences in each set (P<0.001), while comparison between $synDWI_{1000}$ and $conDWI_{1000}$, $synDWI_{1200}$ and $conDWI_{1200}$, $synDWI_{1400}$ and $conDWI_{1400}$ presented no significant differences (p>0.05). Cancer-to-parenchyma contrast ratio almost unchanging showed no significant difference (p>0.05). Both readers showed similar assessments at all DWI sets, with moderate-to-good interobserver agreement (ICC=0.63-0.81, P<0.05).

**Conclusion:** Synthetic DWI is of great comparable image quality and lesion conspicuity in HCC diagnosis versus conventional acquired DWI, while reducing scan time and avoiding the disadvantages of high-b-value DWI.

### SSD 2.8

### LI-RADS ancillary features favoring benignity: is there a role in LR-5 observations?

F. Matteini, R. Cannella, M. Antonucci, D.S. Gagliano, F. Vernuccio, G. Brancatelli; Palermo/IT

**Purpose:** The LI-RADS algorithm allows category downgrade in the presence of ancillary features (AFs) favoring benignity, even in observations categorized as LR-5. This study aims to assess the role of AFs favoring benignity in LR-5 observations and their impact on possible category downgrade.

**Material and methods:** This retrospective study included 119 cirrhotic patients with at least one LR-5 observation imaged with gadoxetate disodium-enhanced MRI between 2016 and 2020. Three readers with different experience levels independently evaluated the presence of AFs favoring malignancy, not HCC in particular, favoring HCC in particular, and AFs favoring benignity. Category downgrade was considered possible in the presence of ≥ 1 AF favoring benignity and no AFs favoring malignancy. Correlation between lesion size and number of AFs was assessed using the Spearman's rank correlation coefficient. The Cohen's kappa (k) test was used to assess inter-reader agreement.

**Results:** Final cohort included 162 LR-5 (mean size: 22.9±15.8 mm). Hepatobiliary phase hypointensity was the most common AF among LR-5 observations (87.7–93.8%). AFs favoring benignity were reported in 9 (5.5%), 20 (12.3%), and 10 (6.2%) observations by R1, R2, and R3, respectively. Category downgrade was considered possible in only one (0.6%) observation by R1 and R3. There was a statistically significant correlation between observation size and number of AFs favoring malignancy (p<0.001), not HCC in particular (p≤0.010), and favoring HCC in particular (p<0.001). The inter-reader agreement ranged from poor to substantial (k=-0.01–0.74).

**Conclusion:** AFs favoring benignity are not uncommon in LR-5 observations but they have a marginal impact for possible category downgrade.

### SSD 2.9

### Role of radiomics in predicting the degree of differentiation of HCC in patients with liver cirrhosis: a preliminary study

E. Damato, M. Di Martino, G. Tatulli, C. Catalano, M. Bezzi; Rome/IT

**Purpose:** The study aimed to identify biomarkers based on clinical patient characteristics, qualitative laboratory data, and CT radiomic features able to non-invasively determine the degree of HCC differentiation.
**Material and methods:** We retrospectively enrolled 47 liver cirrhosis patients with 54 HCC nodules. They were divided into three groups based on the degree of tumour differentiation (G1, G2, G3+G4). Clinical-laboratory data, radiological aspects from CT images, and radiomics data extrapolated using dedicated software were compared to identify significant differences. The non-parametric Mann–Whitney and Pearson's $\chi^2$ tests were used for group comparisons. Parameters with an accuracy >0.85 were considered.
**Results:** Three clinical-laboratory and qualitative parameters (total bilirubin 0.89, lesion diameter 0.858, and albumin 0.85) and six radiomic characteristics ("GLZLM GLNU" 0.91, "GLRLM RLNU" 0.90, "SHAPE Volume vx" 0.889, "GLCM Entropy log10" 0.875, "GLCM_Energy Second Angular momentum" 0.858, and "SHAPE Volume mL" 0.865) were selected. When considered together, GLZLM GLNU and total bilirubin had a diagnostic accuracy to discriminate low-grade (G1+G2) from high-grade (G3+G4) tumours of 0.955 with a sensitivity and specificity of 100% and 83%, respectively. Specifically, high-grade lesions (G3+G4) were more frequently found in patients with total bilirubin levels >1.7 mg/dL and with GLZLM GLNU >120.
**Conclusion:** The radiomic feature GLZLM GLNU associated with the level of total bilirubin may represent a valuable biological marker to accurately identify well- or moderately differentiated HCCs from poorly differentiated or undifferentiated HCCs.

ON DEMAND

*Scientific Session On Demand SSD 3*

### GI Tract Luminal: new trends

### SSD 3.1

### GI stromal tumors: relationship between preoperative CT features and pathologic risk stratification

G. Danti, G. Grazzini, D. Palatresi, F. Fedeli, D. Cozzi, S. Pradella, V. Miele; Florence/IT

**Purpose:** Our retrospective study sought to investigate a relationship between preoperative contrast-enhanced CT (CECT) features of GI stromal tumors (GISTs) and risk of relapse, according to Miettinen's stratified risk classifications.
**Material and methods:** A retrospective analysis was conducted on the preoperative CECT of patients with pathologically proven GIST undergoing surgery between June 2009 and December 2019. Chi-square analysis was used to evaluate the correlation between Miettinen's stratified risk categories and the following imaging features: tumor size and location, growth pattern, margins, type and degree of CE, presence of calcifications, necrosis, signs of ulceration or fistulation, internal hemorrhagic foci, enlarged feeding or draining vessels, ascites, peritoneal implants, lymphadenopathy and/or metastasis.
**Results:** 54 patients (29 men, 25 women) were included in the study for a total of 56 primitive GISTs: 5 (8.9%) no risk, 13 (23.2%) very low risk, 17 (30.4%) low risk, 8 (14.3%) moderate risk, and 13 (23.2%) high risk. Chi-square analysis showed necrosis, ulceration/fistulation, hemorrhage, margins, enlarged vessels, type of CE, and metastasis to be associated with Miettinen's risk categories ($p<0.005$). Logistic regression analysis identified the presence of necrosis and enlarged feeding or draining vessels as predictors of pathologic risk of relapse (overall accuracy of 89.3%).
**Conclusion:** Several CECT features showed a significant correlation with risk of relapse. Preoperative CECT may be helpful in predicting the pathologic risk categories of GISTs, as determined by Miettinen's classification system.

### SSD 3.2

### Magnetization transfer MRI and T2-weighted MR texture analysis for the assessment of intestinal fibrosis in a chronic colitis mouse model

I. De Kock, S. Bos, L. Delrue, P. Hindryckx, M. De Vos, D. Laukens, G.M. Villeirs; Ghent/BE

**Purpose:** To investigate whether magnetization transfer (MT)-MRI and MR texture analysis (MR-TA) can detect and quantify intestinal fibrosis in an established mouse model of fibrosis-inducing chronic colitis.
**Material and methods:** Chronic colitis was caused in 16 C57BL/6 mice following dextran sodium sulphate (DSS) administration during 3 cycles, inducing early-phase inflammation and progressive fibrosis. Regions of interest were drawn over the bowel wall on both 7.0Tesla MT and T2-weighted MR images in each mouse. Textural features (skewness, kurtosis, entropy) were extracted by a filtration histogram technique. Masson's trichrome-stained colon slices were scored to assess inflammation and mural fibrosis. Statistical analysis was performed using mixed model analysis and area under the receiver operating characteristic curve (AUC) analysis.
**Results:** Significant differences between inflammation and fibrosis were found for mean MT ratio (0.288 and 0.580; p= 0.000) and mean TA entropy (4.270 and 4.886; p= 0.012). Mean TA entropy was significantly lower during early-phase inflammation in the first DSS cycle as compared to the last DSS cycle (3.855 versus 4.270; p=0.004) whereas no significant differences in mean MT ratios were observed. High accuracies in differentiating bowel fibrosis from inflammation were found for both MT ratio (AUC= 1.00; p= 0.006) and TA entropy (AUC= 0.93; p= 0.018).
**Conclusion:** MT-MRI and MR-TA can both accurately detect fibrosis in a fibrosis-inducing chronic colitis mouse model. Where TA entropy excels in detecting fibrosis in an inflammatory environment, MT ratio is superior to detect fibrosis as such. MT ratio and TA entropy should be considered as complementary techniques in the detection of intestinal fibrosis.

### SSD 3.3

### Influence of oral contrast type and volume on patient experience and quality of luminal distension at MR enterography in Crohn's disease

G. Bhatnagar[1], S.A. Taylor[2], S. Mallett[2], L. Quinn[3], R. Ilangovan[4], U. Patel[4], A. Jaffer[5], C. Pawley[6], A. Gupta[4], A. Higginson[7], A. Slater[8], D. Tolan[9], I. Zealley[10], S. Halligan[2], T. Metric Trial Investigators[2]; [1]Frimley/UK, [2]London/UK, [3]Birmimgham/UK, [4]Harrow/UK, [5]Guildford/UK, [6]Gloucester/UK, [7]Portsmouth/UK, [8]Oxford/UK, [9]Leeds/UK, [10]Dundee/UK

**Purpose:** To compare the distention quality and patient experience of oral mannitol and polyethylene glycol (PEG) prior to MR enterography.

**Material and methods:** Overall and segmental MRE small bowel distention quality from 105 patients (64 female, mean age 37) recruited to the METRIC trial was rated from 0=poor to 4=excellent by two experienced observers: 68 [65%] received mannitol and 37[35%] received PEG. Additionally, 130 patients (female 77, mean age 34) completed a questionnaire rating tolerability of various symptoms immediately and 2 days after MRE: 85[65%] received mannitol and 45[35%] received PEG. Distension was compared between agents, and between those ingesting ≤1L or >1 L of mannitol using the test of proportions. Tolerability grading was collapsed into "Very tolerable", "Moderately tolerable" and "Not tolerable".

**Results:** Distension quality (per patient) was similar between agents ("excellent" or "good" in 54% [37/68] versus 46% [17/37] with mannitol and PEG, respectively) (percentage difference [95%CI] 8 [-11 to 28]). Jejunal distension was significantly better with mannitol compared to PEG (40% [27/68] versus 14% [5/37] rated as excellent/good, respectively), percentage difference [95%CI] 26 [10 to 42], p=0.005. There was no significant distension difference according to the volume of mannitol ingested. Tolerability was comparable between agents, although fullness following MRE was graded as 'very tolerable' in 44% [37/84] ingesting mannitol versus 27% [12/45] ingesting PEG, difference 17% (95%CI 0.6 to 34%).

**Conclusion:** Oral mannitol-based solutions and PEG generally achieve comparable distension quality and side effect profiles, although jejunal distension is better with mannitol. Neither distension quality nor side effect profile is influenced by the volume ingested.

### SSD 3.4

### Interobserver variation in the interpretation of MR enterography in Crohn's disease

G. Bhatnagar[1], S.A. Taylor[2], S. Mallett[2], L. Quinn[3], R. Beable[4], H.K. Bungay[5], M. Betts[5], R. Greenhalgh[6], A. Gupta[7], R. Hyland[8], A. Higginson[4], R. Ilangovan[7], H. Lambie[8], E. Mainta[6], U. Patel[7], A. Plumb[2], F. Porte[9], H. Sidhu[2], A. Slater[5], D. Tolan[8], I. Zealley[10], S. Halligan[2], T. Metric Trial Investigators[2]; [1]Frimley/UK, [2]London/UK, [3]Birmimgham/UK, [4]Portsmouth/UK, [5]Oxford/UK, [6]Middlesex/UK, [7]Harrow/UK, [8]Leeds/UK, [9]Tunbridge Wells/UK, [10]Dundee/UK

**Purpose:** To evaluate inter-observer variability for the diagnosis and staging of small bowel Crohn's disease using MR enterography (MRE).

**Material and methods:** Datasets from 73 patients (mean age 32, 40 male) recruited to the METRIC trial (28 newly diagnosed, 45 relapsed patients) were each independently read by three radiologists selected from a pool of 20. Radiologists documented the presence and segmental location of small bowel Crohns disease and recorded morphological mural and extramural parameters for involved segments. Agreement for disease presence and extent (i.e. full correct segmental location) against an outcome-based construct reference standard was calculated on a per-patient percentage basis (averaged between pairs of readers). Prevalence-adjusted bias-adjusted kappa (PABAK) was calculated.

**Results:** Overall agreement with the reference standard for small bowel disease presence (new diagnosis) was 68%, ($\kappa$ = 0.36, fair agreement) and 43% for disease extent ($\kappa$ = 0.14, slight agreement), and for relapsed patients 78% ($\kappa$= 0.56, moderate agreement) for presence and 53% for disease extent ($\kappa$=0.07, slight agreement). Overall, all three radiologists correctly agreed with the reference standard for disease presence in 41/59 (69%) of patients with small bowel disease but just 19/59 (32%) for disease extent. Agreement was highest for multi-segment disease, measuring more than 5cm in length with mural thickness >6mm in diameter and increased mural T2 signal.

**Conclusion:** There is a fair and moderate agreement between radiologists for detecting small bowel disease presence using MRE in newly diagnosed Crohn's disease patients and patients with suspected relapse, respectively. Agreement for disease extent is lower.

### SSD 3.5

### The relationship between CT features and histomorphological features and immunohistochemical expression of GI stromal tumors

N. Gunduz, M.B. Dogan, H. Seneldir, O. Ekinci, I.M. Leblebici, O. Alimoglu; Istanbul/TR

**Purpose:** We aimed to evaluate the relationship between the CT features and histomorphological properties and immunohistochemical (IHC) markers.

**Material and methods:** This single-center retrospective study comprised patients with pathologically confirmed GI stromal tumors (STs) in our tertiary referral center. The predefined contrast-enhanced dynamic CT characteristics comprised tumor size, presence of intratumoral cystic openings, hemorrhage and calcification, CT attenuation of solid component (hypo-isodense/hyperdense), and contrast enhancement pattern of solid component (homogeneous/heterogeneous). The GISTs were divided into groups according to NIH risk category, tumor origin, cell type, mitotic count, CD117 and $\alpha$-SMA positivity and Ki-67 index. The frequencies of CT phenotypes were compared between groups.

**Results:** Of 25 GISTs, 16(64%) were gastric and 9(36%) were intestinal in origin. Among CT features, the maximum diameter was higher in epithelioid versus spindled, infiltrative versus expansive, a mitotic count ≥5/50 vs <5/50, necrotic versus non-necrotic, high- versus low-risk category tumors (p<0.05 for all). Only the tumor origin was not related to size. No relationship was found between size and CD-117 or SMA. A heterogeneous enhancement was more frequent in Ki-67 >8 versus Ki-67 <6 tumors (90% vs 46.7%, respectively, p=0.04). The enhancement pattern did not differ according to CD-117 or SMA positivity. Logistic regression analysis revealed that the only independent predictor of a Ki-67 >8 status was the tumor size (OR: 1.02, 95% CI: 1.001–1.046, p=0.04).

**Conclusion:** Heterogeneously enhanced large GISTs at CT imaging strongly suggest the presence of a high Ki-67 index and/or high-risk category. CT features of GISTs do not appear to be associated with diagnostic IHC staining status.

### SSD 3.6

### Performance of CT texture analysis in the response to therapy and survival prediction of advanced gastric cancer after neoadjuvant chemotherapy

M. Zerunian, C. Rucci, M. Polici, B. Bracci, E. Berardi, D. Caruso, A. Laghi; Rome/IT

**Purpose:** To assess the performance of CT texture analysis (CTTA) to predict response to therapy and overall survival in patients with advanced gastric cancer treated with neoadjuvant chemotherapy (n-ChT).
**Material and methods:** Twenty patients with advanced gastric cancer were retrospectively enrolled between December 2015 and February 2019. All participants underwent contrast-enhanced CT at baseline and after n-ChT (FLOT scheme); histology of the specimen after surgical resection was used as the reference standard. An expert radiologist manually draws a volumetric region of interest of the whole tumor before and after n-ChT on CT venous phase at an axial plane. Using a dedicated software (Slicer Radiomics), 109 texture parameters of the first and second order were extracted. Performance of CTTA in the prediction of response to therapy was assessed with receiver operating characteristic (ROC) curve correlated with baseline CT and histologic report; performance of CTTA to predict the overall survival (OS) was tested with ROC curve between CTTA delta (post-nChT and pre-nCHT) and OS. Area under the curve (AUC) with a P<0.05 were considered significant.
**Results:** showed complete response in twelve patients (60%) while partial or non-response was observed in eight patients (40%). Mean OS was 13.5±8.5 months. Among texture parameters, gray-level-non-uniformity showed a significant ROC curve (AUC 0.766, P= 0.03) to predict response to n-ChT. On the other hand, delta CTTA showed significant results for low-gray-level-emphasis (AUC 0.852, P=0.003), long-run-emphasis (AUC 0.926, P<0.001) and autocorrelation (AUC 0.889, P=0.002) to predict OS.
**Conclusion:** CTTA can potentially assume an important supportive role in the radiologic assessment of advanced gastric cancer patients treated with neoadjuvant chemotherapy.

### SSD 3.7

### Local staging of colon tumors with MRI

L. Soydan; Istanbul/TR

**Purpose:** Accurate preoperative staging of colon cancer is important to distinguish between patients who can proceed to upfront surgery and patients who may benefit from neoadjuvant chemotherapy. We evaluated the diagnostic accuracy of preoperative MRI in identifying locally advanced colon cancer, extramural venous invasion (EMVI), a parameter indicating poor prognosis and inter-observer variation of the tumor apparent diffusion coefficient (ADC) values of diffusion-weighted imaging (DWI).
**Material and methods:** 40 patients with colon cancer were evaluated using 1.5 T MRI with T2-weighted imaging, DWI, and contrast enhancement. T-stage, N-stage, EMVI and ADC values of the tumors were assessed. Early tumors were defined as T1 to T3ab (<5mm pericolic invasion) and advanced tumors as T3cd (>5mm extramural invasion) or T4. N+ were nodes with short axis >10mm or >5mm with an irregular border/inhomogeneous signal intensity. EMVI+ was tumor extension to pericolic vessels. MR findings of two readers who were blinded to pathological findings were compared with postoperative histopathological examination which served as reference.
**Results:** Diagnostic accuracy of the two radiologists in staging early versus advanced tumors, N-stage, and detection of EMVI was 88% vs /80%, 60% vs 56%, and 66% vs 60% with an inter-observer agreement of i.0.82 (95% confidence interval [CI]=0.60–1.00), i.0.75 (95% CI=0.35–0.85), and i.0.72 (95% CI=0.20–0.70). All mean ADC values were below 1.0205 $mm^2/s$ , with an intraclass correlation coefficient of 0.78 in T3cd–T4 tumors.
**Conclusion:** Preoperative MRI can distinguish between early and locally advanced colon cancer and may be potentially used to help select patients for neoadjuvant chemotherapy. In advanced tumors, ADC values had high interobserver agreement.

ON DEMAND

*Scientific Session On Demand SSD 4*

### Diffuse liver diseases

### SSD 4.1

### Automated deep learning CT-based liver volume segmentation: defining normal and hepatomegaly for clinical practice

A. Perez[1], V. Noe-Kim[1], M. Lubner[1], R. Summers[2], P.J. Pickhardt[1]; [1]Madison, WI/US, [2]Bethesda, MD/US

**Purpose:** Imaging assessment for hepatomegaly is not well defined and currently utilizes suboptimal unidimensional measures. Liver volume provides a more direct measure for organ enlargement. We applied a validated deep learning artificial intelligence (AI) tool that automatically segments the liver for organ volume and sought to establish thresholds for hepatomegaly.
**Material and methods:** Hepatic volumes were derived for 3065 asymptomatic adults (mean age 54.3 years; 1426M/1639F) who underwent MDCT for colorectal screening (n=1960) or renal donor evaluation (n=1104). Linear regression analysis was utilized to assess major patient-specific determinate(s) of liver volume amongst age/sex/height/weight/BSA. The threshold for hepatomegaly was set at two standard deviations above the mean. Accuracy of craniocaudal and maximal 3D linear measures was assessed. Manual liver volume was compared with automated results in 189 patients.
**Results:** Mean standardized automated liver volume was 1533±375 ml and demonstrated a normal distribution. Patient weight was the major determinant of liver volume, with a linear relationship. From this, a linear weight-based upper limit of normal results in hepatomegaly threshold volume (ml)=14.0(Wt)+979. Linear measures demonstrated only moderate performance for identifying volume-defined hepatomegaly; a craniocaudal threshold of 19 cm was 71% sensitive and 86% specific for hepatomegaly, and a maximal 3D linear threshold of 24 cm was 78% sensitive and 66% specific. For the subset (n=189) with manual versus automated comparison, mean difference in hepatic volume was 2.8% (41 ml).
**Conclusion:** We derived a simple, weight-based threshold for hepatomegaly using an automated liver volume tool. If further validated in larger healthy and diseased cohorts, this approach could provide a more objective measurement of liver size.

### SSD 4.2

### CT portography with esophageal variceal measurements may be used for the evaluation of esophageal variceal severity

S. Wan, B. Song, X. Zhang, Y. He; Chengdu/CN

**Purpose:** To evaluate the severity of esophageal varices (EV) based on CT portography (CTP) measurements of EV in the distal esophagus and to assess whether CTP can be used as a complementary method for endoscopy.
**Material and methods:** A total of 136 EV patients with clinicopathologically confirmed liver cirrhosis were evaluated. All were examined by CTP within 4 weeks of upper endoscopy, patients were divided into a non-conspicuous EV group (mild-to-moderate EV, n=30) and a conspicuous EV group (severe EV, n=106) according to standard endoscopy. The EVD (EV diameter), CSA (cross-sectional surface area of EV), EVV (EV volume), SV (spleen volume) and DLGV (diameter of left gastric vein) were measured independently using 3D-slicer (Boston, USA). These indicators' predictive performances were studied using receiver operating characteristic (ROC) curve analysis, and the area under the curve (AUC), sensitivity and specificity were calculated to distinguish mild-to-moderate from severe EV. Data between the two groups were analyzed by T test or the Mann–Whitney test, a p<0.05 (two-tailed) was accepted as statistically significant for all tests.
**Results:** In those indices, EVD, CSA, EVV and DLGV were larger in the conspicuous group than the non-conspicuous group. The difference between the two groups was statistically significant (p≤0.01). The AUC values of EVD, CSA, EVV and DLGV in differentiating severe EV were 0.729, 0.762, 0.75 and 0.721; sensitivity and specificity of EVD for predicting severe EV were 73.3% and 69.8%, respectively; corresponding values of CSA were 93.3% and 54.7%, EVV were 80% and 65.1%, and DLGV were 83.3% and 53.8%.
**Conclusion:** CTP can be used as a noninvasive method to effectively predict EV severity, which may reduce the invasive examination of endoscopy and be used as a supplementary procedure in cirrhotic patients.

### SSD 4.3

### Quantitative evaluation of liver steatosis with dual-energy on 3rd-generation dual-source CT: comparison with surgical specimen

A. Agostini, M. Tufillaro, A. Borgheresi, L. Ottaviani, R. Rossi, F. Puccio, A. Mandolesi, M. Vivarelli, A. Giovagnoni; Ancona/IT

**Purpose:** To evaluate the accuracy in the quantification of liver steatosis with 3rd-generation dual-source dual-energy CT (dsDECT) with pathology on the surgical specimen as the reference standard.
**Material and methods:** Patients >18 years old, undergoing liver resection between Jan 2018 and Jan 2020, with an abdominal contrast-enhanced dsDECT within 1 month before resection, were retrospectively included. The exclusion criteria were the lack of contrast-enhanced dsDECT or pathological data. The dsDECT examinations were performed at 80–100/150Sn kV and modulated mA, with a triphasic contrast-enhanced protocol (370 mg[I]/ml, 1,3 ml/kg body weight). Liver steatosis was estimated with three-material decomposition algorithm on a dedicated workstation from arterial and venous datasets. Liver attenuations on virtual non-contrast (VNC) from the same datasets were also recorded. Surgical specimens were reviewed by two experienced pathologists in consensus. The estimates of steatosis from dsDECT datasets were correlated with pathology with receiver characteristic curves (ROC) analysis (endpoint: pathological steatosis ≥30%).
**Results:** 48 patients (F/M=18/30, mean age: 65 years old) were included; 15/48 had cholangiocarcinoma while 20/48 were resected for colorectal liver metastases. The dsDECT estimates of steatosis with three-material decomposition achieved significantly higher areas under the curve (AUC 0.872–0.891) than attenuation values on VNC (AUC 0.801–0.813) at ROC analysis; the sensitivity was comparable (84%) while three-material decomposition had higher specificity (86%–91% vs 65–75%).
**Conclusion:** Three-material decomposition has a better diagnostic performance in the quantification of liver steatosis than liver attenuation values on VNC reconstructions.

### SSD 4.4

### Interobserver variability in the evaluation of MR elastography in patients with fibrotic liver disease

M. Raudner[1], D. Bencikova[1], S. Pötter-Lang[1], N. Bastati-Huber[1], I. Shchekoturov[2], S. Kannengiesser[3], M. Krššák[1], A. Ba-Ssalamah[1]; [1]Vienna/AT, [2]Moscow/RU, [3]Erlangen/DE

**Purpose:** To assess the feasibility of a manual segmentation approach in MR elastography (MRE) using a spin-echo echo-planar imaging (EPI) prototype sequence in patients with various hepatic diseases.
**Material and methods:** In total, 111 (46 female, mean age 61.0±13.6 years) individuals were examined at 3T (Magnetom PRISMAfit, Siemens Healthineers, Erlangen, Germany) using a prototype spin-echo EPI sequence. The derived MRE images were independently evaluated by two independent readers, both qualitatively and quantitatively (in kPa) with strict adherence to the QIBA Consensus Profile for MRE of the liver of the Radiological Society of North America.
**Results:** The wave propagation was graded as disorganized in 11 and 9 cases, suboptimal in 34 and 28 cases and optimal in 66 and 74 cases, by both readers, respectively. Mean stiffness was 3.2±1.8 kPa for reader 1 and 3.4±1.8 kPa for reader 2. The agreement of both readers was excellent with an ICC of 0.969 (95%CI 0.950–0.980) with only a minimal bias of -0.14 kPa (95%CI -0,228023 to -0,0532536). There was no correlation between disagreement and increasing kPa ($r_{sp}$=0.019, p=0.856). Also, the mean difference between both readers did not differ between groups with better or worse wave propagation (p=0.549 and p=0.584).
**Conclusion:** Based upon the presented data, assessing the agreement of manual stiffness assessment in MRE at 3T using a spin-echo EPI prototype sequence, the interobserver agreement was excellent. However, evaluating the cause-specific sources for suboptimal wave propagation is warranted.

### SSD 4.5

### Interpretable machine learning for predicting stereotactic body radiation therapy early response in liver colorectal cancer metastasis

S. Waktola, D. Van Der Velden, F. Castagnoli, R.G.H. Beets-Tan; Amsterdam/NL

**Purpose:** To develop a framework for automated early prediction of treatment response after liver stereotactic body radiation therapy (SBRT).
**Material and methods:** 65 patients with pathology-confirmed diagnosis of liver colorectal cancer metastasis and treated with SBRT within our institute, between 2008 and 2020, were retrospectively analysed. The dataset consists of baseline CT (before the start of therapy) and follow-up CT scans (delivered after 4 weeks of therapy). First, all the liver lesions were manually delineated by a radiologist and then categorized into two main classes based on SBRT response: complete response (disappearance of all target lesions), and not responsive (progressive disease). We developed an interpretable machine-learning model by extracting significant features using radiomics and then predicting early response (i.e. before the patient goes to any radiation therapy). To interpret the model predictions, we used model-agnostic explanations (LIME) and Shapley Additive exPlanations (SHAP).
**Results:** During model training, 5-fold cross-validation was performed. Both LIME and SHAP models were able to predict the early SBRT response with a mean AUC of 0.72 and Mann–Whitney U test of P < 0.003.
**Conclusion:** These results indicate that radiomics-based machine-learning models could potentially provide non-invasive biomarkers for early prediction of SBRT treatment response and improving patient stratification for personalized medicine. Interpretable models can pave for better acceptance of machine-learning techniques and to be practically adopted in real clinical settings. However, their evaluation with large and multicenter datasets needs to be researched further.

### SSD 4.6

### Spleen volume as a predictor of hepatic decompensation in patients with chronic liver disease

L. Beer, N. Bastati-Huber, S. Pötter-Lang, J.C. Hodge, Y. Bican, T. Reiberger, M. Mandorfer, A. Ba-Ssalamah; Vienna/AT

**Purpose:** To investigate the accuracy of spleen volume for predicting hepatic decompensation in patients with chronic liver disease (CLD).
**Material and methods:** 402 patients with CLD who had undergone gadoxetic acid-enhanced liver MRI were included. Spleen volumetry was measured either using Syngo-Via software or a 2D-volume-approximation method using only axial and cranio-caudal maximal diameter. Patients were stratified into three groups according to fibrosis stage and present or past hepatic decompensation: non-advanced CLD, compensated-advanced CLD (cACLD), and decompensated-advanced CLD (dACLD). Pearson correlation coefficient assessed the relationship between spleen volume measured by the two above-mentioned methods. The predictive value of spleen volume for first or further hepatic decompensation was investigated using Kaplan–Meier analysis, log-rank tests, and Cox regression analysis.
**Results:** In a subset of 238 patients, we found a strong positive correlation of spleen volume between the 3D-segmentation method and 2D-volume approximation method (R=0.910; p<0.0001). We, therefore, used the simpler and faster approximation method for further analysis. In cACLD patients (n=197), spleen volume was identified as a risk factor for first hepatic decompensation (hazard ratio [HR]: 1.001; 95% confidence interval [CI]: 1.00–1.01, p=0.004) even after the adjustment for known risk factors such as the MELD or albumin levels (adjusted [a]HR: 1.001; 95%CI: 1.00–1.01; p=0.02). In patients with dACLD, spleen volume by trend associated with further hepatic decompensation (HR: 1.00; 95%CI: 1.00–1.01; p=0.052).
**Conclusion:** Spleen volume can predict first hepatic decompensation in patients with compensated-advanced CLD.

### SSD 4.7

### Safe application of the up-to-seven criteria as the upper limit of transplantability in patients with HCC

E. Damato, M. Di Martino, G. Tatulli, P. Lucatelli, M. Bezzi, C. Catalano; Rome/IT

**Purpose:** To evaluate the possibility of extending the liver transplantation criteria in patients with HCC beyond the Milan criteria.
**Material and methods:** Thirty-four cirrhotic patients with HCC were transplanted in our institution using the up-to-seven criteria as the upper limit of transplantability. Results were compared with a similar group of 47 patients who were transplanted using the Milan criteria during the period 2006–2012. HCC recurrence and patient survival rates after liver surgery were analyzed. A comparison between the imaging before liver transplant and explanted liver was also performed.
**Results:** Three- and five-year survival (78.3% vs. 85.2%; 74% vs. 82%) and HCC recurrence (8.5% vs. 5.8%) were similar between the up-to-seven and the Milan criteria groups, respectively. In the up-to-seven group, bigger lesions (p=0.009) and more patients undergoing bridging therapies before liver transplant (p=0.0006) were found. Imaging underestimated more patients in the Milan criteria group (19% vs. 9%), but there was no significant difference.
**Conclusion:** Recurrence and survival in patients transplanted with up-to-seven criteria are acceptable. The up-to-seven criteria enable patients with more advanced HCC to undergo liver transplantation.

### SSD 4.8

### The value of gadoxetic acid-enhanced MR cholangiography in diagnosing dominant strictures in primary sclerosing cholangitis patients

S. Pötter-Lang, N. Bastati-Huber, A. Messner, A. Kristic, G.O. Dovjak, M. Zalaudek, A. Herold, J.C. Hodge, A. Ba-Ssalamah; Vienna/AT

**Purpose:** To compare the diagnostic accuracy of dominant stricture (DS) in primary sclerosing cholangitis (PSC) patients on T2-weighted MRCP, using EASL and AASLD guidelines, with our functional imaging-derived parameter from hepatobiliary-phase (HBP) gadoxetic acid-enhanced T1-weighted MR cholangiography (GA-T1-MRC) using liver function tests (LFT) as the standard of reference.
**Material and methods:** Seventy-four patients (M=42, F=32, mean age 41 years) were given a preliminary diagnosis of DS versus non-DS on T2-weighted MRCP and later HBP GA-T1-MRC by two independent readers, blinded to all patient data. The final diagnosis of DS versus non-DS was based on LFTs, ERCP, and histology, if available. Using societal guidelines, DS was diagnosed on T2-weighted MRCP if common bile duct (CBD) and right or left hepatic main duct diameters were ≤ 1.5 mm and ≤ 1 mm, respectively. We diagnosed DS on GA-T1-MRC if no GA excretion was seen on 20-minute HBP images. The differences between DS and non-DS in both groups and correlation with LFTs and splenic volume were calculated using Mann–Whitney and Spearman tests, respectively.
**Results:** The inter-reader agreement was poor (k< 0.2) for the diagnosis of DS onT2-weighted MRCP, but excellent (k=0.9) for DS diagnosis on HBP-T1-MRC. LFTs and splenic volume were significantly higher in patients lacking excretion compared to those with timely GA excretion on HBP (p=0.001). Our definition of DS in PSC correlated well with functional definition based upon HBP GA-T1-MRC and LFTs (p=0.001), but not with societal guideline-defined measurements (p>0.05).
**Conclusion:** Punctual contrast excretion on HBP GA-T1-MRC is reproducible and correlated well with the presence of DS and LFTs.

ON DEMAND

*Scientific Session On Demand SSD 5*

### Abdominal Oncology

### SSD 5.1

### Associations between perfusion CT variables and immunohistochemical markers of angiogenesis and hypoxia: results from the PROSPECT trial

D. Prezzi[1], V. Goh[1], S. Mallett[2], D. Hill[3], M. Rodriguez-Justo[1], D. Patel[1], S. Khan[1], R. Glynne-Jones[4], V. Boulter[4], S.A. Taylor[1], S. Halligan[1]; [1]London/UK, [2]Prestwood/UK, [3]Birmingham/UK, [4]Northwood/UK

**Purpose:** Hypoxia and angiogenesis are recognised as important drivers of colorectal tumour growth and dissemination and occur early in the adenoma–carcinoma sequence. We explored the biological associations between primary colorectal cancer perfusion CT-derived vascular measurements and immunohistochemistry markers of angiogenesis and hypoxia in the PROSPECT trial.
**Material and methods:** Adult participants with suspected or proven primary colorectal cancer were recruited prospectively from 13 hospitals (ISRCTN 95037585). Exclusions were metastatic disease at staging, prior cancer(s), and contraindications to contrast agents. In addition to standard staging and pathology investigations, participants underwent perfusion CT (blood flow, blood volume) and immunohistochemistry for angiogenesis (CD105, VEGF) and hypoxia (HIF-1, GLUT-1) markers. For CD105 expression, the two areas of highest vascularization (hot spots) were averaged and given as a count per mm2. Scores for VEGF (0–3), Glut-1 (0–8), and HIF-1α (0–6) were based on the intensity and percentage of staining. Correlations between imaging and immunohistochemistry were assessed.
**Results:** 326/448 enrolled participants (226 male; median 67 years (range: 28–92) comprised the final cohort. The majority had locally advanced cancers, ≥T3 stage (227/326, 70%); 151/326 (46%) were node positive. 270/326 tumours underwent further immunohistochemistry. Median CD105 expression was 116 vessels/$mm^2$ (range: 27–393); 254/270 (93%) were VEGF negative; 76/270 (28%) were HIF-1 negative; 114/270 (42%) expressed Glut-1 with 4+ score. No significant associations were noted between perfusion CT and immunohistochemistry variables.
**Conclusion:** In this cohort, there were no biological associations between in vivo CT measurements and immunohistochemistry markers of angiogenesis and hypoxia.

### SSD 5.2

### Prognostic value of pre-operative CT lymph node features and location in stage III colon cancer patients

E.K. Hong[1], F. Landolfi[2], F. Castagnoli[3], R.G.H. Beets-Tan[1]; [1]Amsterdam/NL, [2]Rome/IT, [3]Brescia/IT

**Purpose:** To evaluate the prognostic value of preoperative CT lymph node location and features in stage III colon cancer patients.
**Material and methods:** A total of 176 consecutive stage III colon cancer patients who underwent curative surgery without pre-operative treatment from January 2011 to December 2017 were retrospectively included. The size of the largest lymph node, presence of at least one lymph node with round shape, internal heterogeneity and irregular outer border were assessed on a patient level and the anatomical location (peritumoral, mesenteric and apical) of the lymph node with each imaging feature was recorded and analyzed for prediction of prognosis.
**Results:** Cox regression analysis showed short and long diameters of the largest lymph node, locations of the largest and round-shaped lymph node to be prognostic factors for tumor recurrence (P = 0.004, 0.001, 0.012 and 0.046, respectively). From Kaplan–Meier survival analysis, the 3-year recurrence-free survival (RFS) rates were lower in patients with specific imaging features of lymph nodes than the patients without (66% vs. 90%, P < 0.001 for internal heterogeneity and 77% vs. 90%, P = 0.014 for irregular outer border). The 3-year RFS rate was significantly lower in the group with the largest lymph node located in the apical region than other regions (87%, 78% and 57% for peritumoral, mesenteric and apical, respectively, P = 0.028).
**Conclusion:** Pre-operative radiologic imaging features and location of lymph nodes are predictors of recurrence-free survival in stage III colon cancer patients.

### SSD 5.3

### Diagnostic performance of MRI for staging peritoneal metastases in colorectal cancer patients after neoadjuvant chemotherapy

C.J.V. Rijsemus, N. Kok, A.G.J. Aalbers, M. Engbersen, D. Lambregts, R.G.H. Beets-Tan, M.J. Lahaye; Amsterdam/NL

**Purpose:** MRI can help in selecting colorectal cancer (CRC) patients with peritoneal metastases (PM) for cytoreductive surgery and hyperthermic peritoneal chemotherapy (CRS-HIPEC) by assessing the extent of PM with the peritoneal cancer index (PCI). Several studies have shown an additional value for neoadjuvant chemotherapy (nCHT) before CRS-HIPEC. If CAIRO6, a large multicenter study investigating the added value of nCHT, confirms this, it could affect current guidelines. However, the performance of MRI after nCHT for staging PM in CRC patients has never been investigated. Therefore, the aim is to determine whether DW-MRI can accurately select CRS-HIPEC candidates after nCHT.
**Material and methods:** Patients with PM from CRC and appendiceal origin who received nCHT followed by a diagnostic laparoscopy (DLS) or CRS-HIPEC from January 2016 to August 2020 were eligible. Two radiologists assessed the PCI on DW-MRI (MRI-PCI) after nCHT. The surgical PCI (S-PCI) was subtracted from patient files. The reference standard was histology PCI (H-PCI). The main outcome was the accuracy of DW-MRI after nCHT in predicting whether patients were eligible for CRS-HIPEC.
**Results:** Thirty-three patients were included. Both readers detected all 24 patients (24/33) with resectable disease. Seven out of nine patients with unresectable disease during staging surgery were detected with MRI. The intraclass correlation (ICC) between both readers was excellent (0·89 (0·75 to 0·95)). ICC between S-PCI and MRI-PCI was 0·87 (0·66 to 0·94). S-PCI had similar correlation with H-PCI (0·90 (0·76 to 0·96)) than MRI-PCI (0·89 (0·74 to 0·95)).
**Conclusion:** MRI is a promising tool to re-assess the PCI after neoadjuvant chemotherapy to guide patient selection.

### SSD 5.4

### Spread patterns of colorectal peritoneal metastases

M. Engbersen, C. Rijsemus, T. Buffart, D. Lambregts, A.G.J. Aalbers, N. Kok, R.G.H. Beets-Tan, M.J. Lahaye; Amsterdam/NL

**Purpose:** Right-sided colon tumors with peritoneal metastases (PM) are associated with poorer prognosis than left-sided tumors. Different spread patterns of PM could be a contributing factor. The aim of this study was to explore the natural spread of PM in relation to the primary tumor location on MRI.
**Material and methods:** This was a single-center retrospective cohort study of patients with colorectal PM. Patients with colorectal PM were eligible if they had undergone an abdominopelvic MRI scan following clinical diagnosis of PM. Patients were excluded if they had undergone treatment, local or systemic, between clinical diagnosis and the MRI scan. Patient characteristics, like primary tumor location and the peritoneal cancer index (PCI), were retrieved from patient records and radiological reports. The frequency of affected PCI regions was assessed and compared between tumor sidedness.
**Results:** 126 patients were included with a median age of 65 (IQR: 56-72). 46% percent were male. No difference in mean PCI or number of regions affected was found between patients with a right-sided or left-sided colon tumor (p=0.30 and p=0.44, respectively). PM were found most frequently in close proximity to the primary tumor. In right-sided tumors, small bowel and upper regions were significantly more affected (both p=0.04 ) than in left-sided tumors.
**Conclusion:** Colorectal peritoneal metastases seem to spread from the close proximity of the primary tumor to further abdominal sites via known peritoneal fluid flows. Known unfavorable sites for cytoreductive surgery (small bowel and upper abdominal sites) are, therefore, more frequently reached from right-sided tumors.

### SSD 5.5

### Diagnostic impact of CT slice thickness in detecting high-risk colon cancer

E.K. Hong[1], S.J. Park[2], J.M. Lee[2], R.G.H. Beets-Tan[1]; [1]Amsterdam/NL, [2]Seoul/KR

**Purpose:** To evaluate the diagnostic impact of different slice thickness of CT in optimizing detection of high-risk colon cancer.
**Material and methods:** A total of 42 patients who underwent curative surgical resection without pre-operative treatment of colon cancer was retrospectively included in this study. The portal phase scan of each patient was reconstructed into 1-mm-, 3-mm- and 5-mm-thick images and utilized for analysis. Tumor and lymph node staging of colon cancer were independently performed by 2 radiologists on each set of images. Sensitivity, specificity and area under the receiver operating characteristic curve (AUC) were calculated and compared between the reconstructed images with different slice thicknesses.
**Results:** The mean AUC, sensitivity and specificity of distinguishing tumor invasion beyond muscularis propria were 0.733, 83.3 and 63.3 for 1-mm-, 0.725, 91.7 and 53.3 for 3-mm- and 0.667, 66.7 and 67.4 for 5-mm-thick images. There was a statistically significant difference in diagnostic performances between using 1-mm-, 3-mm- and 5-mm-thick images in detecting over pT3 stage tumors (P = 0.003). For identifying the lymph node involvement of colon cancer, the mean AUC, sensitivity and specificity were 0.550, 56.2 and 53.8 for 1-mm-, 0.526, 43.7 and 61.5 for 3-mm- and 0.538, 50.0 and 58.7 for 5-mm-thick images. There was no statistically significant difference in diagnostic performances for detecting lymph node involvement when using different slice thicknesses.
**Conclusion:** The difference in CT slice thickness resulted in a significant difference in detecting high-T stage colon cancer. However, there was little or no advantage in reducing slice thickness in detecting lymph node involvement of colon cancer.

### SSD 5.6

### Role of CT texture analysis in predicting peritoneal carcinomatosis in patients with gastric cancer

G.M. Masci, F. Iafrate, F. Ciccarelli, D. Grasso, A. Laghi, C. Catalano; Rome/IT

**Purpose:** The aim of the study was to perform CT texture analysis in patients with gastric cancer (GC) to investigate the potential role of radiomics in predicting the occurrence of peritoneal carcinomatosis (PC).
**Material and methods:** In this single-center retrospective analysis, GC patients with and without synchronous PC (group PC and group non-PC, respectively) were enrolled, based on surgically confirmed degree of peritoneal involvement. Pre-operative contrast-enhanced CT examinations were evaluated. Texture analysis was performed on portal phase images: the region of interest was manually drawn along the margins of the primitive lesion on each slice and the volume of interest of the whole tumor was obtained. A total of 38 texture parameters were extracted and analyzed. ROC curves were performed on significant texture features. Multiple logistic regression was conducted on features with the best AUC to identify differentiating variables for both groups.
**Results:** Ninety patients with GC (group PC, n=45; group non-PC, n=45) were included. T1/T2 tumors were prevalent in group non-PC, whilst T3 was significantly associated with group PC. Significant differences were observed for 22/38 texture parameters. Features with the highest AUC in ROC curve analysis were volume and GLRLM_LRHGE (0.737 and 0.734, respectively), which were found to be independent differentiating variables of group PC in the multiple regression analysis (OR 8.44, [95% CI, 1.52–46.8] and OR 18.99 [95% CI, 84–195.31], respectively).
**Conclusion:** Our preliminary results suggest the potential value of CT texture analysis for predicting the occurrence of PC from GC, which may be helpful to stratify patients and address them to the most appropriate treatment.

### SSD 5.7

### Added value of MRI for extraperitoneal findings in potential cytoreductive surgery and hyperthermic intraperitoneal chemotherapy candidates: seeing the whole picture

I. Van 'T Sant, E. Nerad, C.J.V. Rijsemus, M. Engbersen, D. Lambregts, R.G.H. Beets-Tan, A.G.J. Aalbers, M.J. Lahaye, N. Kok; Amsterdam/NL

**Purpose:** In colorectal cancer (CRC) patients, selecting suitable cytoreductive surgery and hyperthermic intraperitoneal chemotherapy (CRS–HIPEC) candidates is based on the location and extent of peritoneal metastases (PM) and presence of extraperitoneal metastases. MRI is increasingly used to accurately assess the extent of PM; however, the significance of extraperitoneal findings (EPFs) in these scans has never been evaluated before.
**Material and methods:** CRC patients with an additional MRI scan after standard workup with CT for preoperative staging between January 2016 and January 2020 were selected. CT and MRI reports were reviewed for new extra-peritoneal findings on MRI (MR-EPF) and MRI findings concerning lesions previously indicated as equivocal (uncertain benign/malignant) on CT. Reference standard was surgical results or follow-up imaging.
**Results:** In 158 included patients, 60 findings (in 58/158 patients) were noted on MRI: twenty-six (43%) were new findings and thirty-four (57%) were equivocal findings on CT. Of the 34 equivocal findings, 27 were 'rejected/less likely malignant' and 7 'confirmed/more likely malignant' based on MRI. In 29 patients (18%), the MR-EPFs could have contributed to a more complete CRS–HIPEC. Three patients (2%), eligible for CRS–HIPEC on CT, were deemed inoperable due to MR-EPFs. Six EPFs were missed on imaging altogether.
**Conclusion:** MRI had an added value in more than a third of the patients due to EPFs that were undetected or indeterminate on CT and could have had a clinical impact on 20% of the patients. Combined with the known accurate detection of peritoneal disease on MRI, MRI seems a logical addition to the diagnostic workup of potential CRS–HIPEC candidates.

### SSD 5.8

### US-guided omental biopsy: diagnostic yield and correlation with CT findings

A. Perez, M. Lubner, P.J. Pickhardt; Madison, WI/US

**Purpose:** The greater omentum is a common site of malignant peritoneal spread and is a useful target for percutaneous biopsy. We evaluated the diagnostic efficacy of percutaneous US-guided omental biopsy, correlated with pre-biopsy CT findings.
**Material and methods:** Inclusion criteria consisted of US-guided biopsy with pre-biopsy CT available for review at a single academic institution. Demographics, clinical, and imaging findings at pre-biopsy CT and US biopsy were evaluated. Cases of CT-guided omental biopsy were also assessed.
**Results:** 163 patients who underwent US-guided omental biopsy were included (mean age, 65±12 years; 120F/43M; mean BMI, 28.9±7.9). On pre-biopsy CT, omental disease appeared infiltrative in 127 (78%) and mass-forming in 36 (22%) cases. Infiltrative soft-tissue component varied from 10% to 100% relative to fat, correlating with mean attenuation (r=0.83; p<0.01), and with hypoechoic (n=105) versus hyperechoic (n=58) US appearance (p<0.01). Mean omental thickness was 2.6±1.2 cm at the biopsy site (range, 0.7–6.7 cm). Biopsies (156 cases 18-gauge core, 7 cases FNA; mean passes 2.5) were diagnostic in 155 (95%) cases. Gynecologic (n=82; 50%) and gastrointestinal (n=45; 28%) malignancies were most common. 106 (65%) cases had extra-omental disease potentially amenable to biopsy. Only eight CT-guided omental biopsies were performed over this period, six (75%) of which followed planned US biopsy (non-visualization>non-diagnostic). No complications of biopsy were reported.
**Conclusion:** US-guided core biopsy of suspected omental disease provides a safe and effective means for tissue diagnosis. Although the typical pattern of omental infiltration often lacks a discrete target at US, tissue sampling of the thickened structure nonetheless yields a diagnosis in the vast majority of cases.

### SSD 5.9

### National adherence to structured reporting of MRI in rectal cancer

G. Alvfeldt[1], L. Blomqvist[2], N. Sellberg[1], P. Aspelin[3]; [1]Solna/SE, [2]Stockholm/SE, [3]Huddinge/SE

**Purpose:** In 2014, a national workshop program and a reporting template and manual for rectal cancer primary staging using MRI were introduced by the National Swedish Colorectal Cancer Registry. The purpose of this study was to identify if there was a gap between the content in MRI reports from 2016 and the template and to explore and compare differences in content in everyday reporting practice. The aim was to examine how reported findings correlate to the content of the national template and manual and if reporting completeness varies based on implementational differences of the template in the Radiology Information System (RIS).
**Material and methods:** 250 MRI reports from 10 hospitals in 4 healthcare regions in Sweden were collected. Reports were analyzed using qualitative content analysis with a deductive thematic coding scheme based on the template and manual.
**Results:** Three distinctive reporting styles were identified with significant variations of content coverage in relation to the template and manual. The relative completeness of everyday reporting practice of rectal cancer staging in relation to the template ranged from 63.9% to 92.9%, based on the different reporting styles.
**Conclusion:** The overall information completeness on a departmental level is closely related to the implementation of the evidence-based template in the RIS. Reporting practice supported by template-based reporting supersedes free-text reporting by far when it comes to information completeness, harmonized terminology, standardized and structured content. The implementation of template-based reporting in the RIS is a key factor to conform to evidence-based practice.

### SSD 5.10

### Initial experience in staging oesophageal/gastro-oesophageal cancer with integrated 18F-fluorodeoxyglucose positron emission tomography/MRI

A.R. Sharkey[1], S. Bhuva[1], B.-R. Sah[1], A. Green[1], S. Jeljeli[1], R. Neji[2], G. Cook[1], V. Goh[1]; [1]London/UK, [2]Frimley/UK

**Purpose:** 18F-fluorodeoxyglucose positron emission tomography (FDG PET)/MRI may improve cancer staging by combining sensitive cancer detection with high-contrast resolution detail. We compare the diagnostic performance of 18F-FDG PET/MRI to 18F-FDG PET/CT in staging primary oesophageal/gastro-oesophageal cancer.
**Material and methods:** Following ethical approval and informed consent, participants with newly diagnosed oesophageal/gastro-oesophageal cancers were enrolled. Participants were excluded if there was prior or concurrent malignancy. Participants received 326±28 MBq 18F-FDG. After 60 minutes' uptake, 18F-FDG PET/CT (GE Discovery 710) was performed, followed by PET/MRI (Siemens mMR) from skull base to mid-thigh. 18F-FDG PET/CT interpretation was performed by two nuclear medicine physicians, and 18F-FDG PET/MRI by a dual-accredited nuclear medicine physician and radiologist, both in consensus and blinded to other findings. Per-patient diagnostic performance was compared, with tumour board decisions (based on standard investigations) set as the reference standard using the McNemar test, with statistical significance set at 5%.
**Results:** 24 participants (22 male; 68.8 ± 9.1 years) were enrolled. 4 participants were excluded as they did not complete the 18F-FDG PET/MRI scan. Compared to the reference standard, the primary tumour was staged concordantly in 100% (20/20) with 18F-FDG PET/MRI, and 80% (16/20) with 18F-FDG PET/CT; nodal metastases in 100% (20/20) with 18F-FDG PET/MRI, and 19 (95%) with 18F-FDG PET/CT; and distant metastases in 100% (20/20) with 18F-FDG PET/MRI, and 95% (19/20) with 18F-FDG PET/CT. There was no significant difference between 18F-FDG PET/CT and 18F-FDG PET/MRI performance in T- (p=0.52), N- (p=0.91), or M-staging (p=0.94).
**Conclusion:** 18F-FDG PET/MRI is not inferior to PET/CT for staging gastro-oesophageal cancers, with the added advantage of being a one-stop investigation.

### SSD 5.11

### Reporting colon cancer staging using a template

M.R. Pedersen, C. Dam, M. Loft, S. Rafaelsen; Vejle/DK

**Purpose:** The purpose of this study was to evaluate the effect of completeness of the radiological reports in primary local staging colon cancer when using a template.
**Material and methods:** The study used primary staging reports retrieved from the departments RIS/PACS. Five key tumour descriptors were evaluated within each report: tumour morphology (polypoid or annular), information on tumour breach of the colon wall (≥ T3), tumour out-growth in mm, nodal status and TNM in conclusion. The failure to provide a description of the presence or absence of a feature in a report counted as ‘not reported’. To allow comparisons between reporting styles, the template or free-text style of reporting was also recorded.
**Results:** During a two-year period, a total of 666 patient CT reports were evaluated at the colorectal center multidisciplinary team (MDT) conference. In 200 of these reports, a template was used. Information on tumour morphology (polypoid or annular) was present in 81% of the template reports vs 9% in free-text style. The figures in percentage for information on tumour breach of the colon wall (≥ T3) were 93% vs 48%, tumour out-growth in mm: 51% vs 17%, nodal status: 99% vs 86% and TNM in conclusion: 98% vs 51%, P < 0.0001.
**Conclusion:** The present study provides additional support for the routine use of template reports to improve imaging reporting standards in colon cancer.

### SSD 5.12

### CT texture analysis of esophageal cancer: changes induced by chemoradiotherapy

R. De Robertis, C. Gasparini, N. Simoni, R. Mazzarotto, M. Pavarana, M. Milella, S. Giacopuzzi, G. De Manzoni, S. Montemezzi; Verona/IT

**Purpose:** Chemoradiotherapy (CRT) has a pivotal role in the treatment of esophageal cancer. The aim of this study was to evaluate changes in tumor volume and CT texture parameters before and after CRT in patients with locally advanced esophageal cancer.
**Material and methods:** CT examinations of patients with esophageal squamous cell carcinoma and adenocarcinoma that underwent CRT between 2012 and 2020 were retrospectively analyzed using LifeX (www.lifexsoft.org). Tumor volume and CT textural features at diagnosis and at the end of CRT were compared using the Wilcoxon test. P values <.05 indicated a statistically significant difference.
**Results:** 47 patients were included in this study (37 males, 10 females; mean age 60 years, age range 41–76 years). A significant tumor volume decrease was found after CRT (30.5 vs 17.2 ml, p<.001). Significant changes were found for 6 conventional indices (HU_min, p=.017; HU_mean, p=.002; HU_Q1, p=.006; HU_Q2, p=.001; HU_Q3, p=.003; HU_skewness, p=.008), 2 shape features (surface, p<.001; compacity, p<.001) and 12 high-order metrics (GLRLM_HGRE, p=.007; GLRLM_SRHGE, p=.013; GLRLM_GLNU, p<.001; GLRLM_RLNU, p<.001; NGLDM_busyness, p<.001; GLZLM_LZE, p=.035; GLZLM_HGZE, p=.038; GLZLM_SZHGE, p=.011; GLZLM_LZLGE, p=.036; GLZLM_LZHGE, p=.031; GLZLM_GLNU, p<.001; GLZLM_ZLNU, p<.001).
**Conclusion:** CT texture analysis can identify tumor changes after CRT other than volume reduction in patients with esophageal cancer.

### SSD 5.13

### Risk of developing gallbladder cancer in patients with gallbladder polyps detected on trans-abdominal US examination: a systematic review and meta-analysis

Z. Riddell[1], B. Willis[2], B. Coles[3], A. Cleves[3], K. Foley[4]; [1]Bridgend/UK, [2]Birmingham/UK, [3]Cardiff/UK, [4]Llantrisant/UK

**Purpose:** This systematic review will determine the risk of gallbladder cancer (GBC) in patients with gallbladder polyps (GBP) detected on trans-abdominal ultrasound (TAUS). If possible, meta-analysis will estimate the effect size of identified prognostic factors for the development of GBC.
**Material and methods:** Primary studies reporting prognostic factors in patients with GBP will be systematically searched from databases including MEDLINE, Embase and Cochrane Library. This review will include observational cohort, cross-sectional and case–control studies. Included studies will have aimed to determine the natural history of gallbladder polyps and/or evaluated the risk of gallbladder malignancy by monitoring a defined cohort of patients. Studies unavailable in the English language will be excluded. Risk of bias will be assessed using the Quality in Prognostic Factor Studies (QUIPS) tool. The strength of the overall weight of evidence will be judged using the Grading of Recommendations Assessment, Development and Evaluation (GRADE) working group methodology. This review has been registered with PROSPERO (CRD42020223629).
**Results:** This systematic review will be reported according to the Preferred Reporting Items for Systematic Reviews and Meta-Analyses (PRISMA) guidance. After abstract screening, data from full-text articles meeting the inclusion criteria will be extracted by two independent observers. If heterogeneity is considerable ($I^2 > 75\%$), then quantitative data synthesis will not be performed.
**Conclusion:** GBP are a common finding on TAUS, but the risk of developing GBC is low. This systematic review will identify important prognostic factors for GBC and will inform the update of ESGAR joint society GBP guidelines.

ON DEMAND

*Scientific Session On Demand SSD 6*
### Acute abdominal conditions and miscellaneous

### SSD 6.1
### Diagnostic impact of American Association for Surgery of Trauma injury scale in abdominal CT scan for the assessment of liver traumatic lesions
C. Maino[1], D. Ippolito[2], T.P. Giandola[2], D. Gandola[2], C. Talei Franzesi[2], M. Ragusi[2], S. Sironi[1]; [1]Milan/IT, [2]Monza/IT

**Purpose:** To determine if clinical and laboratory data can impact the management of patients with traumatic liver lesions according to the American Association for Surgery of Trauma (AAST) scale.
**Material and methods:** All hemodynamically stable patients with traumatic liver injury underwent a contrast-enhanced CT scan to assess and quantify liver damage. Imaging data were evaluated by a general surgeon and a radiologist (both with more than 15 years of experience). The reviewers graded liver lesions according to the AAST scale and, during the first revision session, they were blinded to clinical data. The primary study outcome was to determine patients' management [operative (OM) or not-operative (NOM)] based exclusively on imaging CT findings and, then, by adding laboratory data.
**Results:** A total of 103 patients were retrospectively enrolled. A good agreement was found for AAST grade I, II, III, and V (k= 0.870, k=0.880, k=0.900, and k=1), while in grade IV, the agreement was fair (k=0.455). According to the only first revision section, the accuracy to determine the management was higher for the radiologist (AUC=0.850, 95%CI 0.770–0.950) than the surgeon (AUC=0.700 95%CI 0.550–0.820) achieving a statistically significant difference (p=0.025). During the second revision session, after unmasking clinical and laboratory data, the overall accuracy between the two readers was statistically comparable (AUC=0.880 and AUC=0.850, p>0.05).
**Conclusion:** The CT liver damage score, according to the AAST scale, represents a useful and fast approach to correctly address the management of liver trauma patients, offering reliable information that well correlates with clinical and laboratory data.

### SSD 6.2
### Interobserver agreement and diagnostic accuracy of CT signs of internal hernia in laparoscopic Roux-en-Y gastric bypass patients
M. Lozano Ros, J.R. Olalla-Munoz, J.M. Plasencia Martínez, A. Moreno Pastor, C.M. Ortiz Morales, I.M. González Moreno, J. Trejo Falcón; Murcia/ES

**Purpose:** To assess interobserver agreement and diagnostic accuracy of individual and combined CT signs for the diagnosis of internal hernia (IH) in laparoscopic Roux-en-Y gastric bypass (LRYGB) patients.
**Material and methods:** In our retrospective case–control study, an electronic search of LRYGB patients with a postoperative CT was performed. Cases (n=8) were consecutively recruited, with LRYGB and a surgically confirmed IH (gold standard). Controls (n=24) were randomly selected, with LRYGB and a CT for other indications. Two expert (abdominal and emergency) radiologists and one senior resident randomly and blindly reviewed the CTs, looking for swirl sign, small bowel obstruction (SBO), mushroom sign, clustered loops, hurricane eye sign, small bowel behind the SMA, right-sided jejuno-jejunal anastomosis, edema and ascites. They also registered the overall impression of IH. Interobserver agreement (Kappa index), diagnostic accuracy of individual signs and of three combined models of signs, and OR (95% CI Chi-squared test) of combined models were calculated.
**Results:** Sensitivity and specificity for individual signs were modest (<80%). Interobserver agreement showed considerable variability, yielding the highest results for SBO (K=0.474–0.636), edema (K=0.419–0.538) and swirl sign (K=0.363–0.518). The agreement for overall impression was moderate (K=0.415–0.570), being the highest among experts (K=0.570). Combined models (swirl sign+SBO, swirl sign+SBO+clustered loops, swirl sign+SBO+clustered loops+edema) drastically increased sensitivity (25–100%) and specificity (72.7–100%), being the highest (both 100%) for the abdominal expert; and interobserver agreement, being perfect (K=1) between experts.
**Conclusion:** CT diagnosis of IH in LRYGB patients is complex. Combined models of CT signs can increase diagnostic accuracy and interobserver agreement.

### SSD 6.3
### Correlation of timed barium esophagogram with Eckardt score in primary achalasia patients treated with peroral endoscopic myotomy
J.E. Lee, M.H. Lee, S.-Y. Choi, S. Lim; Bucheon/KR

**Purpose:** The purpose of our study was to evaluate the role of timed barium esophagogram (TBE) in quantitative measurement of improved esophageal emptying in primary achalasia patients treated with peroral endoscopic myotomy (POEM). Also, we investigated its correlation with improvement of clinical symptoms measured with Eckardt score.
**Material and methods:** This retrospective study included 30 patients who underwent POEM due to primary achalasia. All patients underwent TBE as a baseline study, and the clinical status was evaluated with Eckardt score, using presence and frequency of dysphagia, regurgitation, substernal pain, and weight loss. Follow-up evaluation was done 3 months after POEM. Pre- and post-POEM TBE results were compared using a calculated value based on height and width of barium column on 1-, 2- and 5-minute images. Also, its correlation with improvement of Eckardt score was evaluated using Pearson's correlation test.
**Results:** There was a significant decrease in calculated value of the barium column between the pre- and post-POEM TBE studies (654.4 ± 566.8 to 167.1 ± 283.7, p < 0.01). Also, the Eckardt score decreased significantly after POEM (6.7 ± 2.0 to 0.8 ± 1.0, p < 0.01). Pearson's correlation test revealed that there was a positive correlation with improvement of TBE results and Eckardt score (correlation coefficient = 0.391, p = 0.032).
**Conclusion:** TBE is an objective method for quantitative measurement of improved esophageal emptying in primary achalasia patients treated with POEM and shows positive correlation with the clinical symptoms evaluated with Eckardt score.

### SSD 6.4

### Abdominal acute CT findings in COVID-19 patients admitted to the emergency department

F. Pucciarelli, T. Polidori, G. Guido, M. Zerunian, E. Lucertini, D. Caruso, A. Laghi; Rome/IT

**Purpose:** The purpose of this study is to evaluate the frequency and the principal abdominal imaging findings on CT in patients tested positive for severe acute respiratory syndrome coronavirus 2 (SARS-CoV-2) infection, admitted to the emergency department of our Institution.
**Material and methods:** CT scans of patients with symptoms suspicious for SARS-CoV-2 infection admitted to the emergency department of our Institution from March 2020 to December 2020 were retrospectively analyzed. Inclusion criteria were as follows: a) patients tested positive for COVID-19 on real-time reverse transcriptase-polymerase chain reaction (rtPCR); b) patients with abdominal symptoms (pain, vomiting and diarrhea); c) patients that underwent both enhanced and unenhanced chest and abdominal CT scans. CT scans of included patients were examined by two radiologists in consensus to evaluate if acute detectable CT abdominal findings were present.
**Results:** From an initial number of 538 CT, a total of 56 patients (twenty-seven males and twenty-nine females, ages ranging from 27 to 93 years) tested positive for COVID-19 and with abdominal symptoms were analyzed. Abdominal CT findings were found on 32.2% (n=18/56) of CT scans. In particular, CT scans showed pancreatitis 7.1% (n=4/56), cholecystitis 8.9% (n=5/56), small bowel thickening 1.7% (n=1/56), colitis 3.6% (n=2/56), mesenteritis 8.9% (n=5/56) and both pancreatitis and cholecystitis 1.7% (n=1/56). The remaining 67.8% (n=38/56) CT did not show acute intra-abdominal pathology. 21/56 of the patients demonstrated typical lung base findings associated with COVID-19 on CT.
**Conclusion:** Within the SARS-CoV-2 infection spectrum findings, radiologists should consider abdominal symptoms as a possible manifestation of COVID-19 and consider performing an abdominal CT due to moderate abdominal involvement.

### SSD 6.5

### Do coeliac and hepatic artery variations complicate pancreatoduodenectomy? A CT study

N. Gunduz, M.B. Dogan, F. Buyuker, O. Alimoglu; Istanbul/TR

**Purpose:** The coeliac artery branching and hepatic artery variations are not uncommon and may complicate surgery in patients with periampullary cancers. We aimed to assess the prevalence and types of variations of coeliac and hepatic arteries and their relationship with surgical complications in patients undergoing pancreatoduodenectomy.
**Material and methods:** In this single-center study, we retrospectively reviewed the contrast-enhanced abdominal computed tomography scans of the patients with periampullary cancer imaged before surgery. The coeliac artery branching with a particular focus on hepatic artery origin was assessed on arterial phase images.
**Results:** A total of 66 patients [39 (59.1%) males] with a mean age of 64±13 were included. The most frequent tumor origin was pancreatic head in 41 (62.1%) cases, followed by ampulla in 15 (22.7%) cases. Arterial variations were observed in 15 (22.7%) cases. These included 11 cases with hepatic artery (HA) origin variations (right HA originating from superior mesenteric artery in 8, right HA originating from aorta in 1, left HA originating from aorta in 1, both left and right HA originating from coeliac trunk in 1 case) and 4 coeliac trunk variations (one each case from Uflacker types 1, 2, 3 and 5). Total perioperative complications occurred in 14 cases (13 fistulae and 1 hematoma). The higher rate of complications in the variation group [5 (33.3%)] as compared to no-variation [9 (17.6%)] group did not reach statistical significance (p=0.279).
**Conclusion:** The numbers of coeliac and hepatic arterial variations are quite common but do not seem to significantly increase complications in patients undergoing pancreatoduodenectomy.

### SSD 6.6

### Automated segmentation of the stomach on MRI using a MultiRes Unet

T.J. Wright, A. Bard, H. Fitzke, S.A. Taylor; London/UK

**Purpose:** Quantitation of GI motion using MRI shows considerable promise. However, a current limitation is the need for time-intensive manual segmentation of images. We examined the performance of two neural network architectures to determine the feasibility of fully automated gastric segmentation.
**Material and methods:** Datasets from 196 patients undergoing MR enterography and prepared with oral mannitol were collated from an anonymous database. A single frame from a 20-second dynamic balanced gradient echo sequence was automatically selected based on the shortest Euclidean distance to the pixel-wise median over time. The reader manually segmented the stomach wall in the selected frame using Horos (Annapolis, MD USA) that was reviewed by an expert with >8 years' experience and became the ground truth. The performance of two convolutional neural network architectures (Unet and MultiRes Unet) was assessed with three different loss functions (binary crossentropy, binary crossentropy weight balance, and Dice coefficient loss). Two measures of agreement (intersection over union, IOU; Dice coefficient) and two measures of distance (Hausdorff distance, HD; mean contour distance, MCD) were used to test segmentation accuracy against the ground truth.
**Results:** The best performing architecture was a MultiRes Unet with a Dice coefficient loss function, generating metrics of 0.69 IOU, 0.76 Dice, 30.4mm HD, and 5.25mm MCD. However, 15% of individual overlap scores were 0, indicating complete segmentation failure.
**Conclusion:** This method has potential for the automatic segmentation of gastric volumes, but requires optimisation. Larger training datasets, higher quality images, a tailored acquisition protocol, and a user-defined 'seed' point may reduce the number of failed segmentations.

### SSD 6.7

### Patients with small gallbladder polyps: a long-term follow-up study

M.R. Pedersen, P.O. Otto, S.R. Rafaelsen; Vejle/DK

**Purpose:** Gallbladder polyps are lesions that protrude from the inside of the gallbladder wall into the cavity and are generally detected by abdominal ultrasonography. The knowledge about the growth rate of gallbladder polyps < 6 mm is limited, especially in patients without risk factors. The aim of this study was to examine the long-term follow-up growth in gallbladder polyps < 6 mm in size and to explore the risk of developing gallbladder cancer.
**Material and methods:** Abdominal ultrasonography reports from 2007 to 2009 were reviewed, including reports on patients diagnosed with a gallbladder polyp (polyp size < 6 mm) during the 2007–2009 period. The patients were invited to a final follow-up ultrasonography of the gallbladder conducted during October 2019 to February 2020. A total of 154 patients were included (100 women and 54 men).
**Results:** In 53 patients (34.4%), the polyp was not visible at the ultrasonography follow-up. Gallbladder polyps were found in 101 (65.6%) patients. Single polyp was found in 49 patients (31.8%) and 52 (33.8%) patients had multiple polyps. The median polyp size was 4 mm (range: 2.0–5.9 mm) at baseline compared with 4 mm (range: 1.7–15.0 mm) at the follow-up. A total of 15 patients experienced polyp growth of 2 mm or more. None developed gallbladder cancer.
**Conclusion:** Our study showed that gallbladder polyps < 6 mm had a low probability of increasing in size. No cases of gallbladder cancer were observed among the patients. The need for follow-up in patients with small gallbladder polyps is questionable.

### SSD 6.8

### Imaging manifestations of acute GI bleeding: a case-based review with management

U. Gupta, S. Chinnappan, R. Ramachandran, V.S. P.M.; Chennai/IN

**Purpose:** To retrospectively analyse the unexplained causes of hypotension in patients presenting with hematochezia or hematemesis in a non-trauma setting using CT angiography and correlate with digital subtraction angiography (DSA) and endoscopy.
**Material and methods:** A series of cases of acute GI bleed presenting with hypotension in non-traumatic patients were chosen from a period of five years from 2015 to 2020 whose CT angiogram was done in Philips brilliance 16 slice scanner and GE revolution 128 slice CT scanner. The imaging findings were reviewed and correlated with DSA, intraoperative findings and endoluminal procedures like endoscopy and colonoscopy guided ligation.
**Results:** A total of 16 cases were identified and reviewed: spontaneous gastric perforation (n=1); aortogastric fistula (n=1); intramural hematoma of oesophagus following thrombolysis (n=1); oesophageal and gastric varices (n=2); perforated Meckel's diverticulum (n=2); Henoch–Schonlein pupura vasculitis involving small bowel loops (n=3); angiodysplasia of caecum, distal ileum and rectum (n=3); bleeding caecal diverticulum (n=1); graft versus host disease (n=1); arteriovenous malformation of rectum (n=1). The management included medical management (4 out of 16), surgery (3/16), DSA and embolization (3/16) and colonoscopy/endoscopy-guided ligation of bleeder (3/16). 3 patients succumbed to hypovolemic shock before initiation of any definite treatment.
**Conclusion:** Imaging plays an important role in the diagnosis and management of patients with acute GI bleeding. CT angiography is one of the most important diagnostic modalities with easy availability. By diagnosing the cause of acute GI bleeding, we could help guide endoscopic, endovascular or surgical management and appropriate treatment. Radiologist plays a significant role in accurate diagnosis and guides the clinician for definitive management.

### SSD 6.9

### Influence of clinical suspicion on CT accuracy of acute mesenteric ischemia: retrospective study of 362 patients

S. Anglaret[1], A. Dallongeville[1], H. Beaussier[1], C. Touloupas[1], I. Boulay Coletta[1], A.-M. Tardivel[1], S. Béranger-Gibert[1], S. Silvera[1], G. Chatelier[1], M. Ronot[2], M. Zins[1]; [1]Paris/FR, [2]Clichy/FR

**Purpose:** Acute mesenteric ischemia (AMI) may be underdiagnosed when not clinically suspected before CT is performed. We assessed the influence of clinical suspicion of AMI on CT accuracy.
**Material and methods:** This retrospective single-centre study included patients who underwent CT in 2014–2019 and had clinically suspected AMI and/or confirmed AMI. CT protocols were adapted based on each patient's presentation and on findings from unenhanced images. The CT protocol was considered optimal for AMI when it included arterial and portal venous phases. CT protocols, accuracy of reports, and outcomes were compared between the groups with and without suspected AMI before CT.
**Results:** Of the 375 events, 337 (90%) were suspected AMI and 66 (18%) were AMI, including 28 (42%) with and 38 without suspected AMI. These two groups did not differ significantly regarding the medical history, clinical presentation, or laboratory tests. The CT protocol was more often optimal for AMI in the group with suspected AMI (26/28 [93%] vs. 28/38 [74%], p=0.046). Diagnostic accuracy was not different between groups with and without suspected AMI (26/28 [93%] vs. 34/38 [90%], p=1.00). However, it was lower in the group without suspicion of AMI when the CT protocol was not optimal for AMI (27/28 [96%] vs 7/10 [70%], p=0.048).
**Conclusion:** The negative influence of not clinically suspecting AMI can be mitigated using a tailored CT protocol.

### SSD 6.10

### The value of Doppler US in the assessment of patients with abdominal angina before and after endovascular treatment

E. Kopyto, M. Matuszek, M. Kuczyńska, Ł. Światłowski, K. Pyra, M. Sojka; Lublin/PL

**Purpose:** To assess the utility of Doppler US in the initial qualification of patients with clinical symptoms of abdominal angina for endovascular treatment and during postoperative follow-up (FU).
**Material and methods:** 30 patients (23 women, 7 men) with clinical symptoms of abdominal angina and suspected atherosclerotic etiology were included in the study. Each patient was evaluated by US (B-mode, color and spectral Doppler) and CT angiography (CTA), as a reference method. Based on the results of a diagnostic imaging workup, patients were referred for endovascular treatment. FU Doppler US was performed at 4–6 weeks after the intervention.
**Results:** Doppler US examination revealed significant stenoses of the visceral arteries in 27 patients; 15 superior mesenteric arteries (SMAs) and 12 co-existing celiac trunk and SMA narrowings. Doppler US failed to detect visceral stenoses in 3 cases (1 SMA and 2 celiac trunk and SMA stenoses), for which CTA indicated significant stenosis. Each of these patients underwent endovascular treatment (angioplasty with/without stenting). FU Doppler US was performed at 4–6 weeks after intervention and confirmed undisturbed visceral blood flow in 27 patients; 3 patients required CTA due to failure to visualize visceral arteries.
**Conclusion:** Doppler US evaluation constitutes a valuable alternative for CTA both in primary diagnostics and FU of patients with abdominal angina. Based on the study results, Doppler US is characterized by high diagnostic efficacy, and CTA should be required only in individual cases.

### A

Aalbers A.G.J.: SSD 5.3, SSD 5.4, SSD 5.7
Agnello F.: SSD 2.2, SSD 2.6
Agostini A.: **SSD 4.3**
Alimoglu O.: SSD 3.5, SSD 6.5
Alvfeldt G.: **SSD 5.9**
Ambrosetti A.: SSD 1.3, SSD 1.8
Ambrosetti M.C.: SSD 1.3, SSD 1.8
Amodio A.: SSD 1.3
Amthauer H.: SSL 1.2
Ananthakrishnan R.: SSD 1.10
Anglaret S.: **SSD 6.9**
Antonucci M.: SSD 2.8
Ardon R.: SSL 1.4
Aspelin P.: SSD 5.9
Atkinson D.: SSL 3.6
Auer T.A.: SSL 1.2

### B

Ba-Ssalamah A.: SSD 1.9, SSD 2.3, SSD 4.4, SSD 4.6, SSD 4.8, SSL 1.3, SSL 3.4
Baltzer P.: SSD 2.3
Bard A.: SSD 6.6
Bariani M.: **SSD 1.8**
Bastati-Huber N.: SSD 1.9, SSD 2.3, SSD 4.4, SSD 4.6, SSD 4.8, **SSL 1.3**, SSL 3.4
Batch K.: SSL 1.5
Beable R.: SSD 3.4, SSL 3.1
Beaussier H.: SSD 6.9
Beer A.: SSL 1.3
Beer L.: **SSD 4.6**
Beets G.L.: SSL 2.2
Beets-Tan R.G.H.: SSD 4.5, SSD 5.2, SSD 5.3, SSD 5.4, SSD 5.5, SSD 5.7, SSL 2.2
Bell J.: SSL 2.7
Bencikova D.: SSD 4.4
Béranger-Gibert S.: SSD 6.9
Berardi E.: SSD 3.6
Betts M.: SSD 3.4
Bezzi M.: SSD 2.9, SSD 4.7
Bhatnagar G.: **SSD 3.3**, **SSD 3.4**, **SSL 3.1**
Bhuva S.: SSD 5.10
Bican Y.: SSD 4.6
Bickel H.: SSD 2.3
Blomqvist L.: SSD 5.9
Bogveradze N.: **SSL 2.2**
Borgheresi A.: SSD 4.3
Bos S.: SSD 3.2
Bossuyt P.M.: SSL 3.2
Boulay Coletta I.: SSD 6.9, SSL 1.4
Boulter V.: SSD 5.1, SSL 2.4, SSL 2.5
Bracci B.: SSD 3.6
Brancatelli G.: SSD 2.1, SSD 2.5, SSD 2.6, SSD 2.8, SSL 3.3
Brejnebøl M.W.: **SSL 1.6**
Buffart T.: SSD 5.4
Bungay H.K.: SSD 3.4
Buskens C.: SSL 3.2
Buyuker F.: SSD 6.5

### C

Cammà C.: SSD 2.1, SSD 2.5, SSD 2.5
Cannella R.: **SSD 2.1**, **SSD 2.5**, **SSD 2.6**, SSD 2.8
Caruso D.: SSD 3.6, SSD 6.4
Castagnoli F.: SSD 4.5, SSD 5.2
Catalano C.: SSD 2.9, SSD 4.7, SSD 5.6
Causa Andrieu P.I.: **SSL 1.5**
Celsa C.: SSD 2.1, SSD 2.5
Chandramohan A.: **SSD 1.5**, **SSL 2.1**
Chatelier G.: SSD 6.9
Chatoor D.: SSL 3.6
Chen X.-L.: SSL 2.6
Chiang C.L.: SSD 2.4
Chinnappan S.: SSD 6.8
Chiu K.W.H.: SSD 2.4
Choi S.-Y.: SSD 6.3
Chowdhury S.D.: SSD 1.5
Ciccarelli F.: SSD 5.6
Cleves A.: SSD 5.13
Coles B.: SSD 5.13
Comelli A.: SSD 2.5
Cook G.: SSD 5.10, SSL 2.7
Cozzi D.: SSD 3.1
Cronin C.: SSD 1.4

### D

D'Souza R.: SSL 2.1
Dallongeville A.: SSD 6.9, SSL 1.4
Dam C.: SSD 5.11
Damato E.: **SSD 2.9**, **SSD 4.7**
Danti G.: **SSD 3.1**
Dassignies G.: SSL 1.4
De Kock I.: **SSD 3.2**
De Manzoni G.: SSD 5.12
De Reviers A.: SSL 1.4
De Robertis R.: **SSD 5.12**
De Vos M.: SSD 3.2
Deere H.: SSL 2.7
Del Chiaro M.: SSD 1.2
Delaney F.: **SSD 1.4**
Delrue L.: SSD 3.2
Denecke T.: SSL 1.2
Di Martino M.: SSD 2.9, SSD 4.7
Do R.: SSL 1.5
Dogan M.B.: **SSD 3.5**, **SSD 6.5**
Dovjak G.O.: **SSD 2.3**, SSD 4.8
Dräger F.: SSL 1.2

### E

Eapen A.: SSL 2.1
Eibenberger E.: SSL 1.6
Ekinci O.: SSD 3.5
Emmanuel A.: SSL 3.6
Engbersen M.: SSD 5.3, **SSD 5.4**, SSD 5.7

### F

Fedeli F.: SSD 3.1
Fehrenbach U.: **SSL 1.2**
Fenlon H.: SSD 1.4
Fitzke H.: SSD 6.6
Foley K.: SSD 5.13
Froböse K.: SSL 1.2

### G

Gagliano D.S.: SSD 2.8
Galia M.: SSD 2.2, SSD 2.6
Gandola D.: SSD 1.1, SSD 6.1
Gangai N.: SSL 1.5
Gasparini C.: SSD 5.12
Geisel D.: SSL 1.2
Giacopuzzi S.: SSD 5.12
Giandola T.P.: SSD 1.1, SSD 6.1
Giovagnoni A.: SSD 4.3
Giuffrida P.: SSD 2.5
Glynne-Jones R.: SSD 5.1, SSL 2.4, SSL 2.5
Goh V.: SSD 5.1, SSD 5.10, **SSL 2.4**, SSL 2.5, SSL 2.7
Golia Pernicka J.: SSL 1.5
Gollifer R.: **SSL 3.6**
González Moreno I.M.: SSD 6.2
Grasso D.: SSD 5.6
Grazzini G.: SSD 3.1
Grecchi A.: **SSD 1.3**
Green A.: SSD 5.10, SSL 2.7
Green M.: SSL 2.7
Greenhalgh R.: SSD 3.4, SSL 3.1
Grzeda M.: SSL 2.7
Guido G.: SSD 6.4
Gunduz N.: SSD 3.5, SSD 6.5
Gupta A.: SSD 3.3, SSD 3.4
Gupta U.: **SSD 6.8**
Gurney-Champion O.J.: SSD 1.7

### H

Halligan S.: SSD 3.3, SSD 3.4, SSD 5.1, SSL 2.4, SSL 2.5, SSL 3.1
Hartenstein A.: SSL 1.2
He Y.: SSD 4.2
Herold A.: SSD 1.9, SSD 2.3, SSD 4.8, SSL 1.3
Higginson A.: SSD 3.3, SSD 3.4, SSL 3.1
Hill D.: SSD 5.1, SSL 2.4, SSL 2.5
Hindryckx P.: SSD 3.2
Hodge J.C.: SSD 2.3, SSD 4.6, SSD 4.8, SSL 1.3
Hong E.K.: **SSD 5.2**, **SSD 5.5**
Horsthuis K.: SSL 3.2
Huy N.: SSL 1.5
Hyland R.: SSD 3.4

### I

Iafrate F.: SSD 5.6
Ilangovan R.: SSD 3.3, SSD 3.4, SSL 3.1
Ippolito D.: SSD 1.1, SSD 6.1

### J

Jaffer A.: SSD 3.3
Jann H.: SSL 1.2
Jeljeli S.: SSD 5.10
Jeremic B.: SSL 2.2
Jesudason M.R.: SSL 2.1
Jeyakumar A.: **SSD 1.6**
John R.: SSL 2.1

### K

Kakhadze S.: SSL 2.2
Kannengiesser S.: SSD 4.4
Kartalis N.: SSD 1.2
Kashyap R.: SSD 1.10
Khababi N.E.: SSL 2.2
Khan S.: SSD 5.1, SSL 2.4, SSL 2.5
Kim D.: SSL 2.3
Kiran S.: **SSD 1.10**
Klaassen R.: SSD 1.7
Kok N.: SSD 5.3, SSD 5.4, SSD 5.7
Kopyto E.: **SSD 6.10**
Kristic A.: **SSD 1.9**, SSD 2.3, SSD 4.8, SSL 3.4
Krššák M.: SSD 4.4
Kuczyńska M.: SSD 6.10
Kumbhar G.: SSD 1.5

### L

La Grutta L.: SSD 2.2
Laghi A.: SSD 3.6, SSD 5.6, SSD 6.4
Lahaye M.J.: SSD 5.3, SSD 5.4, SSD 5.7, SSL 2.2
Lambie H.: SSD 3.4, SSL 3.1
Lambregts D.: SSD 5.3, SSD 5.4, SSD 5.7, SSL 2.2
Landolfi F.: SSD 5.2
Langs G.: SSL 1.3
Laukens D.: SSD 3.2
Leblebici I.M.: SSD 3.5
Lee J.E.: **SSD 6.3**
Lee J.M.: SSD 5.5
Lee M.H.: SSD 6.3
Li H.: SSL 2.6
Lim S.: SSD 6.3
Lior G.: SSL 1.5
Loft M.: SSD 5.11
Löhr M.: SSD 1.2
Lozano Ros M.: **SSD 6.2**
Lu D.: SSL 3.5
Lubner M.: SSD 4.1, SSD 5.8, SSL 1.1
Lucatelli P.: SSD 4.7
Lucertini E.: SSD 6.4
Lupton K.: SSL 1.5

### M

Maas M.: SSL 2.2
Maino C.: **SSD 1.1**, **SSD 6.1**
Mainta E.: SSD 3.4, SSL 3.1
Mak S.H.: **SSD 2.4**
Malleo G.: SSD 1.8
Mallett S.: SSD 3.3, SSD 3.4, SSD 5.1, SSL 2.4, SSL 2.5, SSL 3.1
Mandolesi A.: SSD 4.3
Mandorfer M.: SSD 4.6
Mansueto G.: SSD 1.3, SSD 1.8
Masci G.M.: **SSD 5.6**
Masih D.: SSL 2.1
Matteini F.: SSD 2.5, **SSD 2.8**
Matuszek M.: SSD 6.10
Maya M.: SSL 1.5
Mazzarotto R.: SSD 5.12
Meima-Van Praag E.: SSL 3.2
Menys A.: SSL 3.6
Messner A.: SSD 1.9, SSD 4.8, **SSL 3.4**
Metric Trial Investigators T.: SSD 3.3, SSD 3.4, SSL 3.1
Midiri F.: SSD 2.2
Midiri M.: SSD 2.6
Miele V.: SSD 3.1
Milella M.: SSD 5.12
Mittal R.: SSL 2.1
Mogl M.: SSL 1.2
Montemezzi S.: SSD 5.12
Moreno Pastor A.: SSD 6.2
Moro C.F.: SSD 1.2
Müller F.C.: SSL 1.6

### N

Naini B.: SSL 3.5
Nederveen A.J.: SSD 1.7, SSD 1.11
Neji R.: SSD 5.10, SSL 2.7
Nelissen J.L.: SSD 1.11
Nerad E.: SSD 5.7
Nielsen Y.J.W.: SSL 1.6
Noe-Kim V.: SSD 4.1, SSL 1.1

### O

Olalla-Munoz J.R.: SSD 6.2
Ortiz Morales C.M.: SSD 6.2
Ottaviani L.: SSD 4.3
Otto P.O.: SSD 6.7
Owczarczyk K.: SSL 2.7

### P

P.M. V.S.: SSD 1.6, SSD 6.8
Paisant A.: SSL 3.3
Palatresi D.: SSD 3.1
Panneerselvam P.: SSD 1.6
Park S.J.: SSD 5.5
Patel D.: SSD 5.1, SSL 2.4, SSL 2.5
Patel U.: SSD 3.3, SSD 3.4, SSL 3.1
Pavarana M.: SSD 5.12
Pawley C.: SSD 3.3
Pedersen M.R.: SSD 5.11, **SSD 6.7**
Penzkofer T.: SSL 1.2
Perez A.: **SSD 4.1**, **SSD 5.8**, **SSL 1.1**, **SSL 2.3**, **SSL 3.7**
Perkonigg M.: SSL 1.3
Pickhardt P.J.: SSD 4.1, SSD 5.8, SSL 1.1, SSL 2.3, SSL 3.7
Pijnappel E.N.: SSD 1.7
Pilato G.: SSD 2.1
Plasencia Martínez J.M.: SSD 6.2
Plumb A.: SSD 3.4
Polanec S.H.: SSD 2.3
Polici M.: SSD 3.6
Polidori T.: SSD 6.4
Porte F.: SSD 3.4, SSL 3.1
Pötter-Lang S.: SSD 1.9, SSD 2.3, SSD 4.4, SSD 4.6, **SSD 4.8**, SSL 1.3, SSL 3.4
Pozzi Mucelli R.: **SSD 1.2**
Pradella S.: SSD 3.1
Prezzi D.: **SSD 5.1**, **SSL 2.5**
Pu H.: SSL 2.6
Pucciarelli F.: **SSD 6.4**
Puccio F.: SSD 4.3
Pyra K.: SSD 6.10

### Q

Quinn L.: SSD 3.3, SSD 3.4, SSL 3.1

### R

Rabiolo L.: SSD 2.2
Rafaelsen S.: **SSD 5.11**
Rafaelsen S.R.: SSD 6.7
Ragusi M.: SSD 1.1, SSD 6.1
Ram T.S.: SSL 2.1
Ramachandran R.: SSD 1.6, SSD 6.8
Raman S.: SSL 3.5
Raudner M.: **SSD 4.4**, SSL 3.4
Reiberger T.: SSD 4.6
Renzulli M.: SSL 3.3
Riddell Z.: **SSD 5.13**
Rijsemus C.: SSD 5.4
Rijsemus C.J.V.: **SSD 5.3**, **SSD 5.7**
Rimola J.: SSL 3.3
Rodriguez-Justo M.: SSD 5.1, SSL 2.4, SSL 2.5
Ronot M.: SSD 6.9, **SSL 3.3**
Rossi R.: SSD 4.3
Rucci C.: SSD 3.6
Runge J.: SSD 1.7, SSD 1.11
Ruppli C.: SSL 1.4

### S

Sah B.-R.: SSD 5.10
Sapena V.: SSL 3.3
Schrauben E.: SSD 1.11
Sellberg N.: SSD 5.9
Seneldir H.: SSD 3.5
Sevilimedu V.: SSL 1.5
Sharkey A.R.: **SSD 5.10**
Shchekoturov I.: SSD 4.4, SSL 1.3, SSL 3.4
Shetty R.: SSD 1.5
Sidhu H.: SSD 3.4, SSL 3.1
Silvera S.: SSD 6.9
Simon B.: SSL 2.1
Simoni N.: SSD 5.12
Simpson A.: SSL 1.5
Singh A.: SSL 2.1
Sinkus R.: SSD 1.11
Sironi S.: SSD 1.1, SSD 6.1
Slater A.: SSD 3.3, SSD 3.4, SSL 3.1
Snijder H.J.: SSL 3.2
Sojka M.: SSD 6.10
Song B.: SSD 2.7, SSD 4.2
Soydan L.: **SSD 3.7**
Spick C.: SSD 1.9
Sreenath G.S.: SSD 1.10
Stoker J.: SSD 1.7, SSD 1.11, SSL 3.2
Struik F.: SSD 1.7
Summers R.: SSD 4.1, SSL 1.1
Świątłowski Ł.: SSD 6.10

### T

Talei Franzesi C.: SSD 1.1, SSD 6.1
Tardivel A.-M.: SSD 6.9
Tatulli G.: SSD 2.9, SSD 4.7
Taubmann O.: SSL 1.6
Taylor S.A.: SSD 3.3, SSD 3.4, SSD 5.1, SSD 6.6, SSL 2.4, SSL 2.5, SSL 3.1, SSL 3.6
Terraz S.: SSL 3.3
Thomas A.: SSD 1.5
Tielbeek J.: SSL 3.2
Tolan D.: SSD 3.3, SSD 3.4, SSL 3.1
Touloupas C.: SSD 6.9
Trejo Falcón J.: SSD 6.2
Troelstra M.: SSD 1.11
Tse J.R.: **SSL 3.5**
Tufillaro M.: SSD 4.3

### V

Valente R.: SSD 1.2
Van 'T Sant I.: SSD 5.7
Van Der Velden D.: SSD 4.5
Van Laarhoven H.W.M.: SSD 1.7
Van Rijn K.: **SSL 3.2**
Van Schelt A.-S.: **SSD 1.11**
Vanderbecq Q.: **SSL 1.4**
Venidiktova D.: SSL 3.4
Vernuccio F.: SSD 2.1, SSD 2.5, SSD 2.6, SSD 2.8
Villeirs G.M.: SSD 3.2
Viola I.: **SSD 2.2**
Vivarelli M.: SSD 4.3

### W

Wagner M.: SSL 3.3
Waktola S.: **SSD 4.5**
Wan S.: **SSD 4.2**
Wassenaar N.: **SSD 1.7**, SSD 1.11
Weber M.: SSD 2.3
Wei Y.: SSD 2.7
Wiedenmann B.: SSL 1.2
Willis B.: SSD 5.13
Wilmink J.W.: SSD 1.7
Withey S.J.: **SSL 2.7**
Wong S.M.: SSD 2.4
Wright T.J.: **SSD 6.6**

### X

Xin S.: SSL 1.2

### Y

Yao S.: **SSD 2.7**
Yezzaji H.: SSL 2.1
Yip C.: SSL 2.7
Yuan Y.: **SSL 2.6**

### Z

Zalaudek M.: SSD 4.8
Zamboni G.A.: SSD 1.3, SSD 1.8
Zarate-Lopez N.: SSL 3.6
Zealley I.: SSD 3.3, SSD 3.4, SSL 3.1
Zerunian M.: **SSD 3.6**, SSD 6.4
Zhang X.: SSD 4.2
Zins M.: SSD 6.9, SSL 1.4
Zulkernine F.: SSL 1.5
